# Identifying the most critical behavioral lifestyles associated with MAFLD: evidence from the NHANES 2017–2020

**DOI:** 10.3389/fendo.2024.1375374

**Published:** 2024-07-25

**Authors:** Sicheng Li, Jiajin Chen, Yuqin Zhang, Shourui Huang, Qing Pan, Dan Tang, Tianjiao Lan, Shichen Bu, Yan Wang

**Affiliations:** ^1^ Xiamen Cardiovascular Hospital of Xiamen University, School of Medicine, Xiamen University, Xiamen, Fujian, China; ^2^ Department of Epidemiology & Biostatistics, School of Public Health, Peking University, Beijing, China; ^3^ Department of Epidemiology and Health Statistics, West China School of Public Health/West China Fourth Hospital, Sichuan University, Chengdu, Sichuan, China; ^4^ Medical Device Regulatory Research and Evaluation Center, West China Hospital, Sichuan University, Chengdu, Sichuan, China; ^5^ Department of Acute Infectious Disease Prevention and Control, Xiamen Center for Disease Control and Prevention, Xiamen, Fujian, China; ^6^ West China School of Public Health and West China Fourth Hospital, Sichuan University, Chengdu, China

**Keywords:** lifestyle exposome, multiple exposure, joint association, WQS, BKMR, PAF

## Abstract

**Background & aims:**

Accumulating studies have demonstrated associations between single lifestyle exposures and metabolic dysfunction-associated fatty liver disease (MAFLD). However, the joint effects of lifestyle exposures remain unclear, hindering the development of targeted prevention and control strategies. We aimed to investigate the joint associations between lifestyle exposomes and MAFLD.

**Methods:**

This study included 5,002 participants from NHANES 2017–2020. Lifestyle exposomes, including sleep duration, metabolic equivalent of task (MET), Healthy Eating Index (HEI)-2015 score, alcohol consumption, and smoke exposure, were identified from questionnaire data. MAFLD was diagnosed by vibration-controlled transient elastography measurements and laboratory data. A logistic regression model and the weighted quantile sum method were used to evaluate the associations of single and joint lifestyle exposomes, respectively, with MAFLD. The population attributable fractions (PAFs) were calculated to assess the population benefits of different intervention strategies.

**Results:**

Per-quartile range increases in sleep duration (OR=0.883, 95% CI: 0.826–0.944), MET (0.916, 0.871–0.963), and HEI-2015 score (0.827, 0.756–0.904) were significantly associated with MAFLD. The joint exposure of sleep duration, MET, and HEI-2015 score was associated with MAFLD (0.772, 0.688–0.865), with the highest weight (importance) for MET (0.526). PAFs revealed greater intervention benefits for sleep and the HEI-2015 when the majority of the population (>5%) had a low MAFLD risk (weak intervention targets), whereas MET was the most efficient intervention strategy when minority populations (≤5%) had a low MAFLD risk (strong intervention targets).

**Conclusion:**

This study demonstrated significant associations between MAFLD and single and joint exposures to sleep duration, MET, and HEI-2015 and identified physical activity as the most important lifestyle factor. Further population benefit analyses may provide evidence and suggestions for population-level interventions.

## Graphical Abstract

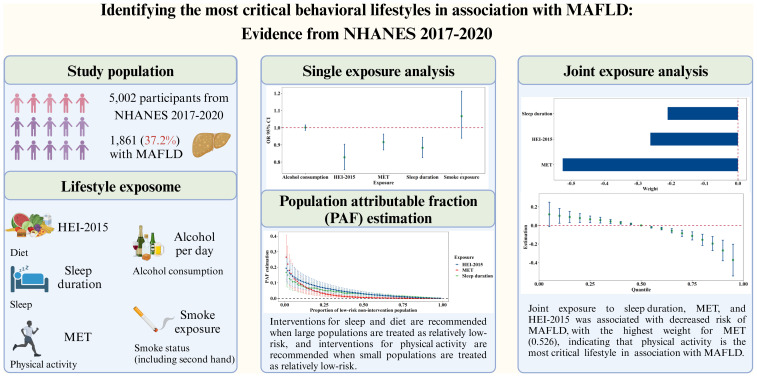

## Introduction

In recent decades, there has been a shift in the understanding of nonalcoholic fatty liver disease (NAFLD), which is now recognized as a hepatic manifestation of a systemic metabolic disorder (1, 2), prompting the renaming of NAFLD to metabolic dysfunction-associated fatty liver disease (MAFLD) (3, 4). In contrast to individuals with NAFLD, individuals with MAFLD face a heightened risk of cardiovascular disease (5) and chronic kidney disease (6) and experience more comorbidities with a less favorable prognosis (7). The global prevalence of MAFLD is approximately 38.8% (8), and MAFLD is associated with increased all-cause mortality (9), imposing a significant socioeconomic burden (10).

Because pharmacological treatment options remain relatively limited, lifestyle interventions remain the bedrock of MAFLD treatment (11). Numerous studies have linked single behavioral lifestyle factors, including smoking, drinking, dietary patterns, physical activity, and sleep (12–18), to MAFLD. However, investigations that solely examine single lifestyle factors without considering their intricate interplay often struggle to determine their relative importance based solely on effect size (19, 20). Similarly, studies on joint lifestyle factors generally assume equal weights for different factors (21). Consequently, current epidemiological studies cannot determine the relative importance of different lifestyle factors in relation to MAFLD, nor can they recommend prioritized lifestyle interventions.

Modern epidemiological studies are increasingly investigating the exposome rather than isolated exposures (22). These exposome-focused approaches help identify critical exposures, leading to findings with broader public health implications (23). Unlike traditional analysis strategies, multiple exposure methods estimate the relative importance of different lifestyle factors and the precise effects of joint lifestyle factors.

Leveraging large-scale data from the National Health and Nutrition Examination Surveys (NHANES), this study investigated the single and joint associations of smoke exposure, alcohol consumption, dietary patterns, physical activity, and sleep with MAFLD. The aim was to estimate the relative importance of these lifestyle factors and their joint effect. The research methods, including study design and participants, assessment of MAFLD, assessment of lifestyles, and statistical analysis, are introduced sequentially. Next, the results, discussion (including comparisons to other studies, interpretations of results, clinical implications, limitations, and strengths), and conclusions are presented.

## Methods

### Study design and participants

**Figure 1:**
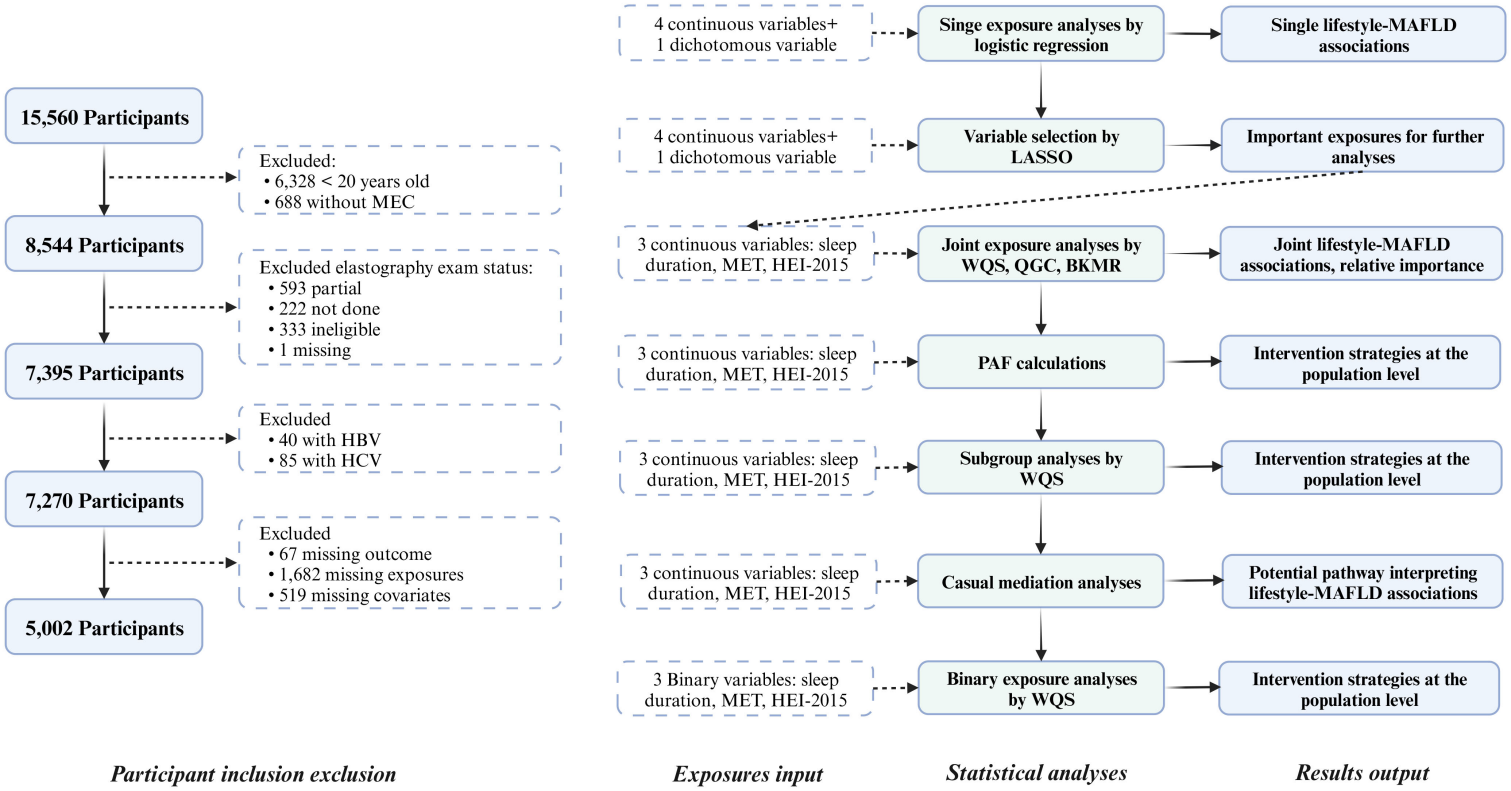
Flow chart of the study participants and statistical analyses. MEC, Mobile examination center; HBV, Hepatitis B Virus; HCV, Hepatitis B Virus; MAFLD, Metabolic-associated fatty liver disease; LASSO, Least absolute shrinkage and selection operator; MET, Metabolic equivalent of task; HEI, Healthy eating index; WQS, Weighted quantile sum; QGC, Quantile G-computation; BKMR, Bayesian kernel machine regression; PAF, Population attributable fraction.

This study included data from the NHANES 2017 to March 2020. Detailed instructions on NHANES 2017–2020 data collection, analysis guidelines, and the full dataset are publicly available (24, 25). Briefly, 15,560 participants aged 0-150 years with data on demographic characteristics, dietary information, physical examinations, laboratory tests, and questionnaires were enrolled. This study excluded individuals who were 1) younger than 20 years, 2) without a mobile examination center (MEC) visit, 3) without complete elastography examination results, 4) with hepatitis B virus (HBV) or hepatitis C virus (HCV) infection, and 5) with missing variables, including 67 missing outcomes, 1,682 missing exposures, and 519 missing covariates (**Figure 1**).

### Assessment of MAFLD

The NHANES 2017–2020 utilized vibration-controlled transient elastography to assess liver fat through the controlled attenuation parameter (CAP) and liver fibrosis through liver stiffness measurement (LSM) (26). Based on fasting time, the number of complete stiffness measurements, and the interquartile range (IQR)/median of liver stiffness, exams were categorized as completed, incomplete, ineligible, or not done. Hepatic steatosis was defined as a CAP ≥285 dB/m (0.8 sensitivity and 0.77 specificity) (27).

MAFLD was defined as the presence of hepatic steatosis with at least one of the following: 1) overweight or obesity (body mass index ≥25 kg/m^2^); 2) diabetes mellitus; or 3) at least two metabolic risk abnormalities. Metabolic risk abnormalities included i) waist circumference ≥102 cm for men and ≥88 cm for women; ii) blood pressure ≥130/85 mmHg or specific drug treatment; iii) triglycerides ≥150 mg/dl; iv) high-density lipoprotein (HDL) cholesterol <40 mg/dl for men and <50 mg/dl for women; v) prediabetes (fasting glucose 100-125 mg/dl or glycated hemoglobin (HbA_1c_) 5.7%-6.4%); vi) homeostasis model assessment of insulin resistance ≥2.5; and vii) high-sensitivity C-reactive protein (HS-CRP) >2 mg/L. Refer to the **Supplementary Methods** for details of the NHANES 2017–2020 variables that were used and how they were calculated.

### Assessment of lifestyles

Diet exposure was determined by the Healthy Eating Index (HEI)-2015, which consists of 13 components (28). The Dietary Approaches to Stop Hypertension diet score, Mediterranean diet score, and dietary inflammatory index were also considered. Sleep exposure was defined as the average sleep duration. Sleep duration level, sleep debt level, sleep difficulty, and daytime sleepiness were defined as dichotomous variables and were integrated into a total sleep score (29, 30). Alcohol exposure was defined as average alcohol consumption (g/day). Heavy alcohol consumption was defined as ≥30 g/day for males and ≥16 g/day for females (31). Smoke exposure was defined as active or passive smoking. Active smokers included current smokers and those who quit smoking. Passive smokers were nonsmokers with serum cotinine concentrations between 0.05 and 10 ng/mL (32). Physical activity was defined as the metabolic equivalent of task (MET). The final MET was calculated from the participant’s type, frequency, and duration of exercise, combined with the recommended MET scores (33).

The main definition of each exposure was used for the main analyses. Alcohol consumption, diet, physical activity, and sleep exposure were included as continuous variables, and smoke exposure was included as a categorical variable in the main analyses. Refer to the **Supplementary Methods** and **Supplementary Tables S1**-**S7** for details of the usage and sources of variables.

### Statistical analysis

Although NHANES 2019–2020 was not expected to be completed due to coronavirus disease 2019 pandemic, NHANES teams created a comprehensive analytic dataset named NHANES 2017–2020. This dataset integrates all existing survey data from 2017 to 2020 and assigns appropriate weights to ensure a nationally representative sample. Given the nonrandom nature of the missing data in this study and the substantial amount of excluded data for variables such as the MEC and elastography exams, it is challenging to determine the overall population to which this analyzed sample corresponds. Consequently, all the results presented in this study pertain exclusively to the original 5,002 participants, and sample weights were not considered.

#### Single-exposure analyses

Logistic regression was employed to estimate the associations between single lifestyle factors and MAFLD. Three models were considered. Model 1: No covariates were included. Model 2: Covariates such as age, sex, education, income, race, and marital status were incorporated. Model 3: Other lifestyle exposures were included as covariates. To provide a visual representation of the analytical framework, a directed acyclic graph is depicted in **Supplementary Figure S1**. Sensitivity analyses included the following: 1) exclusion of participants with cirrhosis (LSM ≥13.1 kPa) to mitigate the risk of reverse causality; 2) implementation of multiple imputation techniques to address missing variables; 3) exploration of alternative definitions for exposures; and 4) consideration of additional covariates, such as general health status, insurance coverage, hypertension, or diabetes.

Furthermore, we calculated E-values to ascertain the significance of potential unmeasured confounding variables necessary to invalidate the reported results (34). The population attributable fractions (PAFs) were calculated for exposures significantly associated with MAFLD to assess the population-level intervention benefits. The PAF is an epidemiologic metric that indicates the percentage of all instances of a specific disease that may be linked to a particular exposure (35). For dichotomous variables, such as smoking status, the PAF was defined as the percentage reduction in MAFLD when the entire population was nonsmokers (in the low-risk group). For continuous variables, this study defined a range of low-risk groups based on population proportions, and a range of PAFs was calculated (36). Refer to the **Supplementary Methods** for details on the E-values and PAFs.

#### Joint exposure analyses

Least absolute shrinkage and selection operator (LASSO) regression was used to select essential lifestyle factors for further joint exposure analyses, complemented by ridge regression as a sensitivity analysis (37). Weighted quantile sum (WQS) (38), quantile G-computation (QGC) (39), and Bayesian kernel machine regression (BKMR) (40) were then used to determine the relative importance of the selected factors and estimate their joint effect. These four approaches are commonly used for multiple-exposure analyses, with the latter three capable of further evaluating the joint effect of multiple exposures.

Briefly, the LASSO method shrinks the coefficients of less important variables closer to zero by adding a penalty term to the loss function, but it cannot further distinguish the relative importance among the selected variables or estimate the joint effect. Like LASSO, ridge regression is incapable of variable selection and is therefore used as a **Supplementary Method**. The WQS method estimates the weights and joint effect of multiple exposures, assuming unidirectionality (positive or negative) for all exposures and linearity for the joint association. The QGC method overcomes the unidirectionality assumption by including additional statistical assumptions, but the linearity assumption remains. The BKMR method estimates nonlinear associations and identifies interactions by introducing high-dimensional reflective surfaces, which are less interpretable. By combining these methods, we can select significant variables, estimate joint effects, overcome the unidirectionality assumption, test linear hypotheses, and confirm the robustness of the outcomes through the consistency of results across methods. The **Supplementary Methods** provide extensive information on the fundamental principles, specific assumptions, and implementation of these techniques. A comparison of the strengths and limitations of these methods can be found in **Supplementary Table S8**. Sensitivity analyses were conducted using various random seeds, except for QGC, as these methods included random sampling, which may have induced variability in the results, i.e., seed dependence (41). Sensitivity analyses were also carried out for WQS and QGC by varying the function parameters.

Subgroup analyses (**Supplementary Methods**) were performed by WQS to determine whether the joint effect of multiple exposures differed across subpopulations (to prioritize interventions for specific subpopulations) and whether the most important exposure differed (to determine specific intervention strategies). QGC was used as a sensitivity analysis method for the subgroup analyses.

Continuous exposure variables were transformed into dichotomous variables according to the recommended cutoff values. A high-quality diet was defined as an HEI-2015 score ≥60 (42), active physical activity was defined as a MET ≥10 h/week (43), and healthy sleep was defined as a sleep duration ≥ 7 h and ≤ 9 h (44). The WQS was used to perform joint exposure analyses of multiple dichotomous variables to determine the priority of interventions in accordance with existing recommendations.

#### Mediation analyses

We conducted causal mediation analyses based on a counterfactual framework (45) to delve into the intricate mechanisms underpinning the association between lifestyle and MAFLD and elucidate the underlying factors contributing to disparities in importance among different lifestyles. We considered the following potential mediators: biological age (46), depression (47), general health status, fasting glucose, systolic blood pressure, diastolic blood pressure, body mass index, HDL, HbA_1c_, HS-CRP, alanine aminotransferase, aspartate aminotransferase, albumin, alkaline phosphatase, and gamma-glutamyl transferase. The details of the mediation analysis and mediator definitions are provided in the **Supplementary Methods**.

We have presented the odds ratio (OR) of MAFLD corresponding to an IQR increase in continuous exposures. All analyses were performed by using R 4.3.1 software. Two-sided tests with P values <0.05 were considered to indicate statistical significance.

## Results

### General characteristics

Among the 5,002 participants included in this study, 1,861 (37.2%) were found to have MAFLD. Notably, individuals with MAFLD displayed distinct demographic and lifestyle traits. Specifically, they tended to be older, were predominantly male, had lower educational attainment, reported shorter sleep duration, engaged in less physical activity, consumed less nutritious diets, and exhibited a greater likelihood of smoke exposure and alcohol use (**Table 1**). Further details regarding the descriptive analyses of the five continuous exposures can be found in **Supplementary Table S9** and **Supplementary Figure S2**.

**Table 1:** The general characteristics of the study population.

Characteristic	Overall (N = 5,002)	MAFLD		*P*-value
		No (N = 3,141, 62.8%)	Yes (N = 1,861, 37.2%)	
Demographic characteristics				
Age, years	50.43 (17.07) **^a^**	48.75 (17.67)	53.26 (15.60)	<0.001
Sex				<0.001
female	2,603 (52.04%)	1,735 (55.24%)	868 (46.64%)	
male	2,399 (47.96%)	1,406 (44.76%)	993 (53.36%)	
Education				<0.001
Less than 9th grade	263 (5.26%)	149 (4.74%)	114 (6.13%)	
9-11th grade	507 (10.14%)	318 (10.12%)	189 (10.16%)	
High school graduate/GED or equivalent	1,144 (22.87%)	686 (21.84%)	458 (24.61%)	
Some college or AA degree	1,749 (34.97%)	1,071 (34.10%)	678 (36.43%)	
College graduate or above	1,339 (26.77%)	917 (29.19%)	422 (22.68%)	
Income				0.612
PLI ≤ 1.3	1,485 (29.69%)	927 (29.51%)	558 (29.98%)	
1.3<PLI ≤ 1.85	756 (15.11%)	465 (14.80%)	291 (15.64%)	
PLI>1.85	2,761 (55.20%)	1,749 (55.68%)	1,012 (54.38%)	
Marital status				<0.001
Married/Living with Partner	2,969 (59.36%)	1,784 (56.80%)	1,185 (63.68%)	
Never married	949 (18.97%)	682 (21.71%)	267 (14.35%)	
Widowed/Divorced/Separated	1,084 (21.67%)	675 (21.49%)	409 (21.98%)	
Race				<0.001
Mexican American	563 (11.26%)	265 (8.44%)	298 (16.01%)	
Non-Hispanic Asian	514 (10.28%)	350 (11.14%)	164 (8.81%)	
Non-Hispanic Black	1,305 (26.09%)	923 (29.39%)	382 (20.53%)	
Non-Hispanic White	1,890 (37.78%)	1,141 (36.33%)	749 (40.25%)	
Other Hispanic	484 (9.68%)	305 (9.71%)	179 (9.62%)	
Other Race - Including Multi-Racial	246 (4.92%)	157 (5.00%)	89 (4.78%)	
Sleep				
Sleep duration, hours	7.72 (1.49)	7.78 (1.49)	7.63 (1.47)	<0.001
Sleep duration level				0.722
Moderate	3,080 (61.58%)	1,940 (61.76%)	1,140 (61.26%)	
Too long or short	1,922 (38.42%)	1,201 (38.24%)	721 (38.74%)	
Sleep debt level				0.014
Low	3,758 (75.13%)	2,396 (76.28%)	1,362 (73.19%)	
High	1,244 (24.87%)	745 (23.72%)	499 (26.81%)	
Sleep difficulty **^b^**				<0.001
No	3,495 (69.89%)	2,284 (72.74%)	1,211 (65.07%)	
Yes	1,506 (30.11%)	856 (27.26%)	650 (34.93%)	
Daytime sleepiness				<0.001
Low	3,710 (74.19%)	2,396 (76.28%)	1,314 (70.65%)	
High	1,291 (25.81%)	745 (23.72%)	546 (29.35%)	
Total sleep score				<0.001
0	81 (1.62%)	44 (1.40%)	37 (1.99%)	
1	463 (9.26%)	255 (8.12%)	208 (11.18%)	
2	1,244 (24.88%)	742 (23.63%)	502 (26.99%)	
3	1,759 (35.18%)	1,121 (35.70%)	638 (34.30%)	
4	1,453 (29.06%)	978 (31.15%)	475 (25.54%)	
Total sleep score level				<0.001
Abnormal	3,547 (70.94%)	2,162 (68.85%)	1,385 (74.46%)	
Normal	1,453 (29.06%)	978 (31.15%)	475 (25.54%)	
Physical activity				
MET, hour/week	76.34 (120.62)	80.39 (123.50)	69.51 (115.32)	<0.001
MET level				<0.001
Inactive	1,760 (35.19%)	1,029 (32.76%)	731 (39.28%)	
Active	3,242 (64.81%)	2,112 (67.24%)	1,130 (60.72%)	
Diet				
HEI-2015	50.47 (12.28)	50.89 (12.50)	49.77 (11.87)	0.004
DASH	26.70 (3.05)	26.82 (3.10)	26.48 (2.96)	<0.001
MED	5.88 (0.94)	5.91 (0.95)	5.84 (0.93)	0.034
DII	1.63 (1.56)	1.60 (1.59)	1.67 (1.51)	0.240
HEI-2015 level				0.052
Low-quality	2,526 (50.50%)	1,553 (49.44%)	973 (52.28%)	
High-quality	2,476 (49.50%)	1,588 (50.56%)	888 (47.72%)	
Smoke				
Smoking status				<0.001
Never	2,859 (57.16%)	1,860 (59.22%)	999 (53.68%)	
Former	1,262 (25.23%)	701 (22.32%)	561 (30.15%)	
Current	881 (17.61%)	580 (18.47%)	301 (16.17%)	
Secondhand smoke status				0.076
Yes	618 (12.36%)	408 (12.99%)	210 (11.28%)	
No	4,384 (87.64%)	2,733 (87.01%)	1,651 (88.72%)	
Smoke exposure status				0.008
Yes	2,761 (55.20%)	1,689 (53.77%)	1,072 (57.60%)	
No	2,241 (44.80%)	1,452 (46.23%)	789 (42.40%)	
Alcohol				
Alcohol consumption, g/day	5.87 (15.17)	5.76 (14.52)	6.06 (16.21)	0.019
Alcohol consumption level				0.425
Excessive	305 (6.10%)	185 (5.89%)	120 (6.45%)	
Moderate	4,697 (93.90%)	2,956 (94.11%)	1,741 (93.55%)	
Mafld associated variables				
CAP, dB/m	265.50 (62.32)	226.86 (39.04)	330.73 (33.02)	<0.001
LSM, kPa	5.87 (4.78)	5.31 (4.28)	6.83 (5.39)	<0.001
BMI, kg/m^2^	29.97 (7.24)	27.54 (6.15)	34.06 (7.11)	<0.001
GLU, mg/dl	113.47 (38.01)	105.47 (27.49)	127.21 (48.29)	<0.001
HbA_1c_, %	5.85 (1.12)	5.62 (0.85)	6.23 (1.38)	<0.001
Waist, cm	100.92 (16.75)	94.44 (14.34)	111.79 (14.75)	<0.001
SBP, mmHg	124.28 (18.75)	122.64 (19.06)	127.01 (17.89)	<0.001
DBP, mmHg	74.82 (11.24)	73.36 (10.95)	77.26 (11.30)	<0.001
Triglyceride, mg/dl	111.83 (106.22)	92.42 (94.21)	145.19 (116.94)	<0.001
HDL, mg/dl	53.50 (15.83)	56.92 (16.14)	47.76 (13.46)	<0.001
HOMA-IR	4.48 (9.50)	3.03 (7.51)	6.98 (11.77)	<0.001
HS-CRP, mg/L	3.92 (6.82)	3.21 (6.51)	5.11 (7.16)	<0.001

**^a^**: Continuous variables are presented as the mean (SD), and discrete variables are presented as n(%).

**^b^**: Missing data exist, as only the variables in **Figure 1** were considered in the exclusion of missing data.

MAFLD, Metabolic-associated fatty liver disease; PLI, Poverty level index; MET, Metabolic equivalent of task; HEI, Healthy eating index; DASH, Dietary approaches to stop hypertension; MED, Mediterranean diet; DII, Dietary inflammation index; CAP, Controlled attenuation parameter; LSM, Liver stiffness measure; GLU, Fasting glucose; HbA_1c_, glycated haemoglobin; SBP, Systolic blood pressure; DBP, Diastolic blood pressure; HDL, High-density lipoprotein; HOMA-IR, Homeostasis model assessment of insulin resistance; HS-CRP, High-sensitive c-reactive protein; SD, standard deviation.

A total of 2,268 (31.2%) participants with missing variables were sequentially excluded, including 1,473 missing HEI-2015 scores, 803 missing income, 249 missing smoke exposure status, 82 missing sleep duration, 67 missing MAFLD diagnosis, nine missing education, and seven missing marital status (**Supplementary Figure S3**). A comparison of the general characteristics between the entire population and the complete population is presented in **Supplementary Table S10**. Notably, disparities in education, race, sleep difficulty, alcohol consumption, smoking status, and waist circumference were statistically significant, suggesting that the missing data might not be missing completely at random.

### Single-exposure analyses

**Table 2** presents the associations between five lifestyle exposures and MAFLD. In Model 3, we observed negative associations of sleep duration (OR=0.883, 95% CI: 0.826–0.944), MET (0.916, 0.871–0.963), and HEI-2015 score (0.827, 0.756–0.904) with MAFLD. Conversely, smoke exposure (1.067, 0.939–1.213) and alcohol consumption (1.001, 0.985–1.016) showed positive associations with MAFLD, but these associations were not statistically significant. Our sensitivity analyses (**Supplementary Tables S11**-**S15**) further confirmed the robustness of the associations of the HEI-2015 score, MET, and sleep duration with MAFLD in terms of direction and statistical significance.

**Table 2:** Associations between single lifestyle exposure and MAFLD.

Exposure	Model 1 ^a^		Model 2 ^b^		Model 3 ^c^	
	OR 95% CI	*P*	OR 95% CI	*P*	OR 95% CI	*P*
**Sleep duration**	0.889 (0.884, 0.936)	<0.001	0.894 (0.843, 0.948)	<0.001	0.883 (0.826, 0.944)	<0.001
**MET**	0.945 (0.910, 0.980)	0.002	0.973 (0.897, 0.979)	0.003	0.916 (0.871, 0.963)	0.001
**HEI-2015**	0.881 (0.813, 0.954)	0.002	0.823 (0.753, 0.900)	<0.001	0.827 (0.756, 0.904)	<0.001
**Smoke exposure ^d^**	1.122 (1.019, 1.236)	0.019	1.073 (0.959, 1.202)	0.217	1.067 (0.939, 1.213)	0.318
**Alcohol consumption**	1.007 (0.994, 1.020)	0.278	0.999 (0.985, 1.014)	0.895	1.001 (0.985, 1.016)	0.967

**^a^**: N=5002, raw results without covariate.

**^b^**: N=5002, adjusted for age, sex, education, PLI, marital status, and race.

**^c^**: N=5002, adjusted for age, sex, education, PLI, marital status, race, and other lifestyle variables.

**^d^**: Smoke exposure was a dichotomous variable with an OR corresponding to the association from no smoking exposure to smoking exposure with MAFLD, and the remaining exposures were continuous variables with an OR corresponding to the association of per IQR exposure increase with MAFLD.

MAFLD, Metabolic-associated fatty liver disease; HEI, Healthy eating index; MET, Metabolic equivalent of task; OR, odds ratio; CI, confidence interval; PLI, Poverty level index.

**Figure 2:**
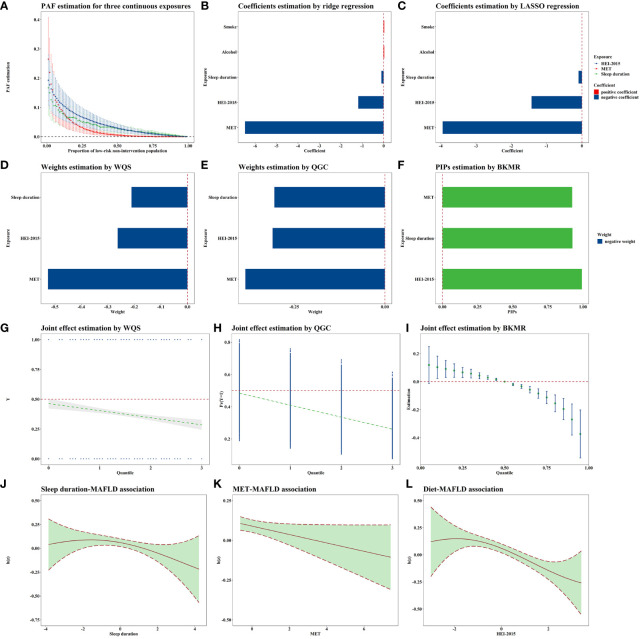
Joint association of lifestyle exposome with MAFLD. All these models were adjusted for age, sex, education, PLI, marital status, race, and all lifestyle variables. N=5,002. **(A)** shows the PAF results for the variables significantly associated with MAFLD in the single-exposure analysis, i.e., sleep, MET, and HEI. The PAFs for sleep and HEI are greater when the quantile is larger, and the PAFs for MET are greater when the quantile is smaller. **(B, C)** demonstrates the results of the penalty algorithm, and it can be found that both LASSO and ridge regression suggest that sleep, MET, and HEI are more important. Combined with the single-exposure analysis strategy results, this study selected the above three variables for subsequent multi-exposure analysis. **(D–F)** demonstrates the order of importance of the three exposures in the joint exposure analysis strategies. **(D, E)** suggests that the MET is the most important, and **(F)** suggests that the HEI is the most important, but the gap between the MET and the HEI is smaller. **(G–I)** shows the joint exposure results, with all three methods suggesting that the risk of MAFLD decreases as the joint exposure increases. **(J–L)** shows the approximately linear exposure–response curves for the three exposures obtained via BKMR. MAFLD, Metabolic-associated fatty liver disease; PAF, Population attributable fraction; LASSO, Least absolute shrinkage and selection operator; WQS, Weighted quantile sum; QGC, Quantile G-computation; BKMR, Bayesian kernel machine regression MET, Metabolic equivalent of task; HEI, Healthy eating index; PLI, Poverty level index.

The E-values for sleep duration, MET, HEI-2015 score, smoke exposure, and alcohol consumption were 1.33, 1.26, 1.43, 1.22, and 1.02, respectively. The PAF results are presented in **Figure 2A**. Notably, as the reference proportion increased, the PAFs for the HEI score and sleep showed an upward trend when the reference proportion was close to 1. Conversely, when the reference proportion approached 0, the PAF associated with MET exhibited a rapid increase. The highest PAF for MET occurred when the reference proportion was less than or equal to 0.05.

### Multiple exposure analyses

Both ridge and LASSO regression analyses revealed that MET had the most substantial protective effect against MAFLD (corresponding to an IQR increase), as depicted in **Figures 2B, C**. Since the variable selection conducted by LASSO excluded smoke exposure and alcohol consumption, neither of which showed statistical significance in the single-exposure analyses, we included smoking and alcohol consumption as covariates in the subsequent multiple-exposure analyses.

According to the WQS analysis (**Figure 2D**), the estimated weights (importances) for sleep duration, MET, and the HEI-2015 score were 0.211, 0.526, and 0.263, respectively. According to the QGC analysis (**Figure 2E**), the estimated weights were 0.305, 0.385, and 0.310, further highlighting the importance of physical activity as a critical lifestyle exposure. When considering the joint effects of sleep duration, MET, and the HEI-2015 score, a negative association with MAFLD was observed in both the WQS (0.772, 0.688–0.865, **Figure 2G**) and QGC (0.710, 0.645–0.782, **Figure 2H**) analyses. The parameters in BKMR nearly converged after 100,000 iterations (**Supplementary Figure S4**). The posterior inclusion probabilities, an indicator of relative importance, of the BKMR for sleep duration, MET, and the HEI-2015 score were 0.925, 0.923, and 0.992, respectively (**Figure 2F**). Joint exposure to sleep duration, MET, and the HEI-2015 score were consistently negatively associated with MAFLD, as illustrated in **Figure 2I**. Notably, the exposure–response curves for sleep duration, MET, and HEI-2015 with respect to MAFLD appeared to be approximately linear (**Figures 2J–L**). Sensitivity analyses conducted with different random seeds (**Supplementary Figures S5**, **S6A**, **S7**, **S8**) and parameters (**Supplementary Figure S6B**, **Supplementary Table S16**) confirmed the robustness of these results.

The subgroup analysis results are shown in **Table 3**. The protective joint association of multiple exposures was notably stronger among individuals with a college degree or higher than those with less than a 9th-grade education. Similarly, Mexican Americans displayed a stronger protective association than non-Hispanic Black and White individuals. Higher exposure weights for HEI scores were observed for females, individuals with a poverty level index between 1.3 and 1.85, and those with a college degree or higher. Conversely, sleep duration had a more pronounced impact on individuals with a high school graduate or equivalent education level. This trend was also observed among individuals of various racial backgrounds, excluding non-Hispanic Black and other Hispanic individuals. However, subgroup sensitivity analyses revealed unstable results (**Supplementary Table S17**). When categorical variables were included in the analysis, we found that weights in the WQS were highest for the MET (0.592), followed by the HEI-2015 score (0.299) and sleep duration (0.110).

**Table 3:** Joint associations of sleep duration, diet, and physical activity exposure with MAFLD within different subgroups using WQS.

Group	N ^a^	Weight estimation			Joint association		*P* for subgroup comparisons
		Sleep	MET	HEI	OR 95% CI	*P*	
**Whole population ^b^**	5,002	0.211	**0.526 ^c^**	0.263	0.772 (0.689, 0.865)	<0.001	—
Sex ^d^							
Female	2,603	0.349	0.220	**0.431**	0.784 (0.654, 0.941)	0.009	Ref
Male	2,399	0.074	**0.537**	0.389	0.745 (0.636, 0.871)	<0.001	0.672
Age							
≥60 years	1,765	0.199	**0.620**	0.181	0.712 (0.574, 0.883)	0.002	Ref
<60 years	3,237	0.353	**0.547**	0.100	0.718 (0.622, 0.829)	<0.001	0.946
PLI							
PLI=1.3	1,485	0.314	**0.534**	0.152	0.770 (0.594, 0.997)	0.047	Ref
1.3<PLI ≤ 1.85	756	0.061	0.448	**0.491**	0.725 (0.524, 1.001)	0.051	0.766
PLI>1.85	2,761	0.265	**0.393**	0.342	0.724 (0.614, 0.854)	<0.001	0.696
Education							
Less than 9th grade	263	0.039	**0.695**	0.266	1.148 (0.635, 2.075)	0.648	Ref
9-11th grade	507	0.339	**0.436**	0.225	0.817 (0.525, 1.272)	0.372	0.367
High school graduate/ GED or equivalent	1,144	**0.450**	0.394	0.156	0.619 (0.470, 0.817)	0.001	0.064
Some college or AA degree	1,749	0.192	**0.610**	0.197	0.801 (0.666, 0.963)	0.018	0.255
College graduate or above	1,339	0.396	0.201	**0.403**	0.510 (0.400, 0.651)	<0.001	0.013
Race							
Mexican American	563	**0.438**	0.226	0.336	0.618 (0.431, 0.884)	0.008	Ref
Non-Hispanic Asian	514	**0.453**	0.321	0.226	0.548 (0.366, 0.821)	0.004	0.666
Non-Hispanic Black	1,305	0.189	**0.414**	0.397	0.866 (0.652, 1.151)	0.324	0.147
Non-Hispanic White	1,890	**0.522**	0.096	0.383	0.857 (0.723, 1.017)	0.077	0.106
Other Hispanic	484	0.140	**0.547**	0.313	0.589 (0.367, 0.945)	0.028	0.877
Other Race - Including Multi-Racial	246	**0.490**	0.477	0.034	0.848 (0.499, 1.439)	0.540	0.331

**^a^**: N denotes the sample size in the model corresponding to each population.

**^b^**: Adjusted for age, sex, education, PLI, marital status, race, and all lifestyle variables.

**^c^**: The most critical exposure in each model is bolded and underlined.

**^d^**: Adjusted for all covariates in **b** except grouped variables, for example, sex.

MAFLD, Metabolic-associated fatty liver disease; WQS, Weighted quantile sum; MET, Metabolic equivalent of task; HEI, Healthy eating index; OR, Odds ratio; CI, Confidence interval; PLI, Poverty level index.

### Mediation analyses

Diastolic blood pressure mediated the associations of sleep duration (proportion mediated, 16.6%, *P*=0.003), MET (14.9%, *P*=0.002), and the HEI-2015 score (15.6%, *P*<0.001) with MAFLD. General health status, HDL, HS-CRP, and gamma-glutamyl transferase mediated the associations between MET and MAFLD and between HEI-2015 and MAFLD. Fasting glucose and triglycerides mediated the association between MET and MAFLD. HbA1c mediated the association between sleep duration and MAFLD. Depression, systolic blood pressure, and body mass index mediated the associations between HEI-2015 scores and MAFLD. More details are provided in **Supplementary Table S18**.

## Discussion

To our knowledge, this may be the first epidemiological study to estimate the relative importance of lifestyle factors and investigate their joint association with MAFLD. Using cross-sectional data from the NHANES 2017–2020, we found that longer sleep duration, higher MET, higher HEI-2015 scores, and joint exposure were associated with a decreased risk of MAFLD. We further identified physical activity as the most critical lifestyle factor on the continuous exposure IQR scale and proposed potential intervention strategies.

### Comparison to other studies

Previous studies have explored the associations between single lifestyle exposures and MAFLD (12–16, 33). As an example, Vilar‐Gomez et al. investigated the relationship between physical activity and NAFLD and reported that the risk of NAFLD was lower in physically active participants than in inactive participants, consistent with our findings regarding the direction and significance of this association (33). Additionally, some studies have considered interactions between single lifestyle exposures. Sun et al., for instance, investigated the joint effects (interaction in reality) of dietary and sleep patterns on the risk of MAFLD and observed a synergistic effect (15).

Furthermore, previous studies have incorporated multiple lifestyle exposures into a one-dimensional score and investigated its associations with other diseases (17, 21, 48, 49). The American Heart Association proposed the Life’s Essential 8 (LE8) score to quantify cardiovascular health, which includes five health behaviors (nicotine exposure, physical activity, diet, body mass index, and sleep health) and three health factors (blood lipids, blood pressure, and blood glucose). Lower LE8 scores were found to be associated with a greater risk of MAFLD (17, 21). However, assuming equal weights for each health metric in LE8 may not align with reality, as the impact of different risk factors on health outcomes can vary significantly (50). Inspired by the work of Kim et al. (51), we jointly analyzed lifestyle exposures and identified physical activity as the most influential lifestyle factor.

### Interpretation of results

According to the logistic regression analysis, the HEI-2015 score had the strongest effect, and the MET had the weakest impact. In Model 3, which included all covariates, the OR for the HEI-2015 score was 0.827 per IQR increase, indicating a 17.3% reduction in MAFLD risk. Further PAF analyses revealed that the PAFs for sleep and the HEI-2015 score were greater when large populations were regarded as relatively low risk (weak intervention targets), whereas the PAF for MET was greater when small populations were regarded as relatively low risk (strong intervention targets). For instance, with the proportion of the low-risk population set at 40%, the PAF for the HEI-2015 score was 4.13%, indicating a 4.13% reduction in MAFLD cases if the entire population’s HEI-2015 score was adjusted by intervention into the range corresponding to the HEI-2015 score of 40% of the population with lower risk. According to the WQS and QGC analyses, MET had the highest weight. In the WQS analysis, the MET, HEI-2015 score, and sleep duration were assigned values of 0, 1, 2, and 3, respectively, according to quartiles, and their joint exposure was also evaluated with values of 0, 1, 2, and 3, with a weight of 0.526 for the MET and an OR of 0.772 for the joint exposure. This translates to a 22.8% reduction in MAFLD risk for each one-unit increase in joint exposure, with MET contributing 52.6% to the reduction. Essentially, a one-unit increase in the MET is linked to a 12.7% reduction in the risk of developing MAFLD.

The inconsistency in the above results may be attributed to the differing statistical assumptions underlying the various methods employed. Logistic regression relies on linear assumptions and does not account for the interplay between different lifestyle exposures. Solely comparing effect sizes while overlooking complex associations between exposures fails to provide actionable intervention guidance. In contrast, PAF analyses offer insights into the proportion of the population at lower risk, thus relaxing linear assumptions. While WQS and QGC also operate on linear assumptions, they consider associations between different lifestyles, providing a more nuanced perspective. In summary, physical activity has emerged as the most critical lifestyle factor associated with MAFLD at the individual level. At the population level, when intervention intensity is low, we recommend focusing on dietary and sleep interventions. Conversely, when the intervention intensity is high, prioritizing physical activity interventions is advisable.

In subgroup analyses, we observed a stronger effect of joint exposure among highly educated individuals and Mexican Americans than among those with less education and non-Hispanic Whites. Although the exact underlying mechanism remains unclear, we hypothesize that unmeasured lifestyle factors might explain this disparity. In the context of promoting health equity, further research is required to explore measures aimed at enhancing the health of vulnerable groups, ultimately working toward achieving universal health. Additionally, this study identified the most critical exposures for each subgroup. However, it is essential to note that the results exhibited some instability when different statistical methods were applied. Given the relatively small sample size (246 in the smallest subgroup), these findings should be interpreted with caution.

In the categorical multiple-exposure analysis, the present study revealed that in terms of WQS weight, MET > HEI-2015 > sleep duration. Taking MET > HEI-2015 as an example, this result implies that an intervention for all those with a MET less than 10 to greater than or equal to 10 would provide greater benefits than an intervention for all those with a HEI-2015 less than 60 to greater than or equal to 60. If the costs of these two interventions are the same, the choice of interventions targeting physical activity is more desirable.

### Clinical implications

This study provides further evidence on lifestyle and MAFLD, showing that increasing physical activity, improving diet, and increasing sleep duration can reduce the risk of MAFLD. Existing studies have demonstrated that lifestyle improvement is still essential for those with MAFLD. On the one hand, for clinical interventions of individuals, patients with or at high risk for MAFLD can be encouraged to actively improve these three lifestyle factors to improve their prognosis or reduce their risk of developing the disease, with a prioritization for physical activity interventions when available (11, 52). On the other hand, for policy interventions for the population, slight improvements in sleep and diet can cause great benefits, and the benefits of extensive improvements in physical activity are greater. Government agencies can further conduct cost–benefit analyses to determine the optimal intervention strategy. The results of PAF analysis and categorical multiple-exposure analysis in this study only represent the effect of a single lifestyle intervention, and subsequent studies should estimate the effect of multiple-exposure joint interventions combined with health economics methods to provide the best recommendations for joint intervention strategies.

### Limitations and strengths

This study has several limitations. First, this is a cross-sectional study, which does not allow for determination of a causal effect and carries the risk of causal inversion. We reduced this risk by excluding patients with cirrhosis and reached consistent conclusions, and prospective cohort studies are needed to validate the causality. Second, approximately 31% of our data were missing but not necessarily completely random, limiting the sample size and representativity. However, we mitigated this issue by multiple imputations and found that the impact of missing data was limited. Finally, we adjusted for confounders according to the DAG and conducted extensive sensitivity analyses. However, some unmeasured confounders might still be present, for which we calculated the E-values. Except for alcohol consumption, the remaining results are unlikely to be overturned.

This study also has several strengths. First, this is the first study to regard lifestyles as an exposome, integrate a range of analytical protocols for intervention-oriented analyses, provide a paradigm for subsequent similar studies, and identify physical activity as the most critical lifestyle factor associated with MAFLD. Second, we calculated PAFs for continuous lifestyle exposures, which were then included in the joint exposure analyses as categorical variables. Different intervention strategies have been proposed and evaluated. Third, the joint use of multiple exposure methods, extensive sensitivity analyses, and robust conclusions drawn are methodological strengths of this study.

## Conclusion

In conclusion, this study found significant associations of single and joint exposures to sleep duration, MET, and HEI-2015 with MAFLD and identified physical activity as the most important lifestyle factor. Future works include 1) conducting longitudinal cohort studies to refine the most important lifestyle factor under causal associations and 2) performing cost–benefit analyses on individual and joint lifestyle interventions to determine the final intervention strategy. 

## Data availability statement

Publicly available datasets were analyzed in this study. This data can be found here: https://wwwn.cdc.gov/nchs/nhanes/continuousnhanes/default.aspx?Cycle=2017-2020. 

## Ethics statement

The studies involving humans were approved by NHANES Institutional Review Board (IRB). The studies were conducted in accordance with the local legislation and institutional requirements. The participants provided their written informed consent to participate in this study. 

## Author contributions

SL: Conceptualization, Methodology, Software, Writing – original draft. JC: Conceptualization, Writing – review & editing. YZ: Methodology, Writing – review & editing. SH: Writing – review & editing. QP: Writing – review & editing. DT: Writing – review & editing. TL: Methodology, Writing – review & editing. SB: Methodology, Writing – review & editing. YW: Funding acquisition, Writing – review & editing.
