# Supplementary material for: Identifying the most critical behavioral lifestyles associated with MAFLD: evidence from the NHANES 2017–2020
Source: Front Endocrinol (Lausanne). 2024 Jul 25;15:1375374. doi: 10.3389/fendo.2024.1375374 (PMC11307443; doi:10.3389/fendo.2024.1375374)
Supplement: Supplementary file 1 [file Presentation_1.pdf]

Supplementary Materials for

Identifying the most critical behavioral lifestyles associated with MAFLD: Evidence from NHANES 2017-2020

Sicheng Li[1], Jiajin Chen[1], Yuqin zhang[2], Shourui Huang[3], Qing Pan[4], Dan Tang[5], Tianjiao Lan[6], Shichen Bu[1], Yan Wang[1]

[1] Xiamen Cardiovascular Hospital of Xiamen University, School of Medicine, Xiamen University, Xiamen, Fujian, China

[2] Department of Epidemiology & Biostatistics, School of Public Health, Peking University, Beijing, China

[3] Department of Epidemiology and Health Statistics, West China School of Public Health / West China Fourth Hospital, Sichuan University, Chengdu, Sichuan, China

[4] Medical Device Regulatory Research and Evaluation Center, West China Hospital, Sichuan University, Chengdu, Sichuan, China

[5] Department of Acute Infectious Disease Prevention and Control, Xiamen Center for Disease Control and Prevention, Xiamen, Fujian, China

[6] West China School of Public Health and West China Fourth Hospital, Sichuan University, Chengdu, China

S. Li, and J. Chen contributed equally

Correspondence:

Sicheng Li, Xiamen Cardiovascular Hospital of Xiamen University, School of Medicine, Xiamen University, No. 2999 Jinshan Road, Xiamen, 361006, Fujian, China. E-mail: lisicheng1027@foxmail.com

Yan Wang, Xiamen Cardiovascular Hospital of Xiamen University, School of Medicine, Xiamen University, No. 2999 Jinshan Road, Xiamen, 361006, Fujian, China. E-mail: wy@medmail.com.cn

Contents

Supplementary methods 4

Exposure variables 4

Sleep 4

Table S1 Assessments of sleep characteristics 4

Diet 5

Table S2 HEI-2015 component and total scores for exemplary menus10 6

Physical activity 7

Table S3 Assessments of physical activity characteristics 8

Alcohol consumptiom 9

Table S4 Assessments of alcohol consumption characteristics 9

Smoke 10

Table S5 Assessments of smoke characteristics 10

Outcome and covariates 11

MAFLD 11

Table S6 Assessments of MAFLD characteristics 12

Table S7 Assessments of covariates and other variables 13

Mediators 14

Biological age 14

Directed acyclic graph (DAG) 15

Statistical methods 15

E-value 15

Population attributable fraction (PAF) 16

Ridge and Least Absolute Shrinkage and Selection Operator (LASSO) regression 17

Weighted Quantile Sum (WQS) 18

Quantile G-computation (QGC) 19

Subgroup analysis 19

Bayesian Kernel Machine Regression (BKMR) 20

Casual mediation analysis 21

Supplementary figures 25

Figure S1 The directed acyclic graph (DAG) of this study 25

Figure S2 Distribution and correlation of five continuous exposures 26

Figure S3 Distribution of missing data and its patterns 27

Figure S4 Traceplot of parameters in BKMR 28

Figure S5 Sensitivity analysis of ridge and LASSO with different random seeds 29

Figure S6 Sensitivity analysis of WQS (random seeds) and QGC (different quantile) 30

Figure S7 Sensitivity analysis of PIPs of BKMR with different random seeds 31
Figure S8 Singe exposure-MAFLD associations with different random seeds 32

Supplementary tables 33

Table S8 A comparison of the six statistical methods used in the manuscript 33
Table S9 Statistical description of continuous exposure variables 34
Table S10 Impact of missing data on population characteristics..35
Table S11 Sensitivity analyses for sleep-MAFLD association 38
Table S12 Sensitivity analyses for PA-MAFLD association...39
Table S13 Sensitivity analyses for diet-MAFLD association 40
Table S14 Sensitivity analyses for smoke-MAFLD association 41
Table S15 Sensitivity analyses for alcohol-MAFLD association 42
Table S16 Sensitivity analyses for WQS using different parameters combinations 43
Table S17 Joint associations of sleep duration, diet, and physical activity exposure with MAFLD within different subgroups using QGC 47
Table S18 Mediation effect of multiple mediators in lifestyle-MAFLD association..............48

Supplementary methods

Exposure variables

Sleep

The definitions of the original and derivative variables used in the manuscript in this section and how they were calculated are shown in Table S1.

Table S1 Assessments of sleep characteristics

Original variable	Code	Assessment	Format	Abbreviation
Usual sleep time on weekdays or workdays	SLQ300	What time (do you/does SP) usually fall asleep on weekdays or workdays?	'HH:MM' ('00:00' to '23:30')	ST_w
Usual wake time on weekdays or workdays	SLQ310	What time (do you/does SP) usually fall asleep on weekends or non-workdays?	'HH:MM' ('00:00' to '23:30')	WT_w
Usual sleep time on weekends	SLQ320	What time (do you/does SP) usually wake up on weekdays or workdays?	'HH:MM' ('00:00' to '23:30')	ST_f
Usual wake time on weekends	SLQ330	What time (do you/does SP) usually wake up on weekends or non-workdays?	'HH:MM' ('00:00' to '23:30')	WT_f
Ever told doctor had trouble sleeping?	SLQ050	(Have you/Has SP) ever told a doctor or other health professional that (you have/s/he has) trouble sleeping?	Yes/No	TS
How often feel overly sleepy during day?	SLQ120	In the past month, how often did (you/SP) feel excessively or overly sleepy during the day?	'Never' to 'Almost always - 16-30 times a month'	OS

Derivative variable	Definition	Format	Reference
Sleep duration	$[(WT_w - ST_w)*5+(WT_f - ST_f)*2]/7$	h	1
Sleep duration level	sleep duration <7 h or >9 h — 0<br>sleep duration ≥7 h and ≤9 h — 1	-	1
Sleep debt	$\lvert (WT_w - ST_w) - (WT_f - ST_f) \rvert$	h	1
Sleep debt level	*Sleep debt* ≥2 — 0; *Sleep debt* <2 — 1	-	1
Sleep difficulty	TS = Yes — 0; TS = No — 1	-	2
Over sleepy	OS ≥5 — 0; OS <5 — 1	-	1
Total sleep score	*Sleep duration level* + *Sleep debt level* + *Sleep difficulty* + *Total sleep score*	-	2

Diet

As there are no discernible differences between HEI-2020 and HEI-2015 in adults[3], we have opted to mainly utilize HEI-2015 for our analyses. HEI-2015 was explicitly designed to assess adherence to the 2015-2020 US Dietary Guidelines for Americans and investigate the relationship between food quality and health-related outcomes[4]. The evaluation comprises thirteen components assessed based on energy-adjusted food and nutrient intakes. Among these thirteen elements, nine gauge the sufficiency of the diet, encompassing aspects such as total fruit consumption, whole fruit intake, total vegetable consumption, greens and beans consumption, whole grain intake, dairy intake, total protein food consumption, and fatty acid intake. The remaining four components evaluate the extent of moderation in the diet, with a focus on refined grains, salt, added sugar, and saturated fats. Concerning the adequacy components, higher scores correlate positively with increased intake levels. Conversely, higher scores correlate negatively with reduced consumption levels for the moderation components. The scoring system incorporates seven components, each evaluated on a scale ranging from 0 to 10, while the remaining six components are assessed on a scale from 0 to 5. Combining these components yields the overall score with a maximum value of 100 points. A higher score signifies a higher-quality diet and a greater adherence to the dietary guidelines outlined in the 2015-2020 Dietary Guidelines for Americans.

Approaches to stop hypertension (DASH) diet score[5], Mediterranean (MED) diet score[6], and dietary inflammatory index (DII)[7] were also considered. Notably, some of the original DII variables, such as eugenol, garlic, ginger, onion, trans fat, turmeric, green/black tea, flavan-3-ol, flavones, flavonols, flavonones, anthocyanidins, ioflavones, pepper, thyme/oregano, rosemary are not included because they are not available in NHANES. Thus, the results of DII need to be viewed with caution.

R package ***dietaryindex*** was used to calculate all these dietary indexes based on data from the two-day dietary surveys[8]. The results of this package were shown to be consistent with the NIH SAS code[9].

Table S2 HEI-2015 component and total scores for exemplary menus[10]

Component	Maximum points[1]	Standard for maximum score	Standard for minimum score of zero
Adequacy			
Total Fruits[2]	5	≥0.8 cup equiv. per 1,000 kcal	No Fruit
Whole Fruits[3]	5	≥0.4 cup equiv. per 1,000 kcal	No Whole Fruit
Total Vegetables[4]	5	≥1.1 cup equiv. per 1,000 kcal	No Vegetables
Greens and Beans[4]	5	≥0.2 cup equiv. per 1,000 kcal	No Dark Green Vegetables or Legumes
Whole Grains	10	≥1.5 oz equiv. per 1,000 kcal	No Whole Grains
Dairy[5]	10	≥1.3 cup equiv. per 1,000 kcal	No Dairy
Total Protein Foods[6]	5	≥2.5 oz equiv. per 1,000 kcal	No Protein Foods
Seafood and Plant Proteins[6,7]	5	≥0.8 oz equiv. per 1,000 kcal	No Seafood or Plant Proteins
Fatty Acids[8]	10	(PUFAs + MUFAs)/SFAs ≥2.5	(PUFAs + MUFAs)/SFAs ≤1.2
Moderation			
Refined Grains	10	≤1.8 oz equiv. per 1,000 kcal	≥4.3 oz equiv. per 1,000 kcal
Sodium	10	≤1.1 gram per 1,000 kcal	≥2.0 grams per 1,000 kcal
Added Sugars	10	≤6.5% of energy	≥26% of energy
Saturated Fats	10	≤8% of energy	≥16% of energy

(1) Intakes between the minimum and maximum standards are scored proportionately.
(2) Includes 100% fruit juice.
(3) Includes all forms except juice.
(4) Includes legumes (beans and peas).
(5) Includes all milk products, such as fluid milk, yogurt, and cheese, and fortified soy beverages.
(6) Includes legumes (beans and peas).
(7) Includes seafood, nuts, seeds, soy products (other than beverages), and legumes (beans and peas).
(8) Ratio of poly- and monounsaturated fatty acids (PUFAs and MUFAs) to saturated fatty acids (SFAs).

Abbreviations: PUFAs:polyunsaturated fatty acids. bMUFAs¼monounsaturated fatty acids. cSFAs¼saturated fatty acids.

Physical activity

The Global Physical Activity Questionnaire (GPAQ) incorporates many components of physical activity, including intensity, duration, and frequency, and it assesses three domains in which physical activity is performed (work-related activity, transportation-related activity, and leisure time-related activity). The GPAQ is administered during the household interview, and participants are asked to identify moderate- and vigorous-intensity aerobic physical activity they participated in during the past 30 days. The original variables and their definitions needed to calculate the MET are shown in Table S3, and the MET at the hourly scale is equal to PAD615*PAQ610*8/60 + PAD630*PAQ625*4/60 + PAD645*PAQ640*4/60 + PAD660*PAQ655*8/60 + PAD675*PAQ670*4/60.

We used 10 MET-hours as the cut-off value in the dichotomous exposure analysis. MET is assigned a value of 1 if it is greater than 10 MET-hours and 0 if it is less than 10 MET-hours[11].

Table S3 Assessments of physical activity characteristics

Original variable	Code	Assessment	Definition	MET Scores
Number of days vigorous work	PAQ610	In a typical week, on how many days (do you/does SP) do vigorous-intensity activities as part of (your/his/her) work?	1 to 7 — valid<br>Refused — NA<br>Don't know — NA<br>Missing — 0	8
Minutes vigorous-intensity work	PAD615	How much time (do you/does SP) spend doing vigorous-intensity activities at work on a typical day?	10 to 840 — valid<br>Refused — NA<br>Don't know — NA<br>Missing — 0	
Number of days moderate work	PAQ625	In a typical week, on how many days (do you/does SP) do moderate-intensity activities as part of (your/his/her) work?	1 to 7 — valid<br>Refused — NA<br>Don't know — NA<br>Missing — 0	4
Minutes moderate-intensity work	PAD630	How much time (do you/does SP) spend doing moderate-intensity activities at work on a typical day?	10 to 900 — valid<br>Refused — NA<br>Don't know — NA<br>Missing — 0	
Number of days walk or bicycle	PAQ640	In a typical week, on how many days (do you/does SP) walk or bicycle for at least 10 minutes continuously to get to and from places?	1 to 7 — valid<br>Refused — NA<br>Don't know — NA<br>Missing — 0	4
Minutes walk/bicycle for transportation	PAD645	How much time (do you/does SP) spend walking or bicycling for travel on a typical day?	10 to 840 — valid<br>Refused — NA<br>Don't know — NA<br>Missing — 0	
Days vigorous recreational activities	PAD655	In a typical week, on how many days (do you/does SP) do vigorous-intensity sports, fitness or recreational activities?	1 to 7 — valid<br>Refused — NA<br>Don't know — NA<br>Missing — 0	8
Minutes vigorous recreational activities	PAD660	How much time (do you/does SP) spend doing vigorous-intensity sports, fitness or recreational activities on a typical day?	10 to 480 — valid<br>Refused — NA<br>Don't know — NA<br>Missing — 0	
Days moderate recreational activities	PAQ670	In a typical week, on how many days (do you/does SP) do moderate-intensity sports, fitness or recreational activities?	1 to 7 — valid<br>Refused — NA<br>Don't know — NA<br>Missing — 0	4
Minutes moderate recreational activities	PAD675	How much time (do you/does SP) spend doing moderate-intensity sports, fitness or recreational activities on a typical day?	10 to 600 — valid<br>Refused — NA<br>Don't know — NA<br>Missing — 0	

Alcohol consumptiom

Alcohol exposure was mainly defined as the average daily alcohol consumption in the past year, derived from the frequency of alcohol consumption in the past year and the average number of drinks per occasion. The original variables used in the computation are shown in the Table S4. Average daily alcohol consumption (g) is equal to ALQ121* ALQ130*14/365[12]. Heavy alcohol consumption was identified as ≥30 g/day for males and 16g for females[13].

Table S4 Assessments of alcohol consumption characteristics

Original variable	Code	Assessment	Definition
Past 12 mo how often drink alcoholic bev	ALQ121	During the past 12 months, about how often did (you/SP) drink any type of alcoholic beverage? PROBE: How many days per week, per month, or per year did (you/SP) drink?	Never in the last year —— 0<br>Every day —— 365<br>Nearly every day —— (365+182)/2=274<br>3 to 4 times a week —— 3.5*52=182<br>2 times a week —— 2*52=104<br>Once a week —— 1*52=52<br>2 to 3 times a month —— 2.5*12=30<br>Once a month —— 1*12=12<br>7 to 11 times in the last year —— (7+11)/2=9<br>3 to 6 times in the last year —— (3+6)/2=5<br>1 to 2 times in the last year —— 2<br>Refused —— NA<br>Don't know —— NA<br>Missing —— 0
Avg # alcoholic drinks/day - past 12 mos	ALQ130	During the past 12 months, on those days that (you/SP) drank alcoholic beverages, on the average, how many drinks did (you/he/she) have? By a drink, I mean a 12 oz. beer, a 5 oz. glass of wine, or one and a half ounces of liquor.)	1 to 7 — valid<br>15 drinks or more — 15<br>Refused — NA<br>Don't know — NA<br>Missing — 0

Smoke

Smoke exposure was defined as active or passive smoking. Active smokers included current smokers and those quit smoking. Passive smokers are non-smokers with serum cotinine concentrations between 0.05 and 10 ng/mL[14]. The original variables used in the computation are shown in the Table S5.

Table S5 Assessments of smoke characteristics

Original variable	Code	Assessment	Definition
Do you now smoke cigarettes?	SMQ040	(Do you/Does SP) now smoke cigarettes?	Every day —— 1 (current)<br>Some days —— 1 (current)<br>Not at all —— 1 (quit)<br>Refused —— NA<br>Don't know —— NA<br>Missing —— 0 (never)
Cotinine, Serum (ng/mL) [a]	LBXCOT	Cotinine, Serum (ng/mL)	0.011 — invalid (below lower detection limit)<br>0.011 to 1620 — valid<br>Missing —— NA

[a]: The half-time of cotinine among the general population is about 16 hours[15]. Thus, the passive smoke variable only reflects recent passive smoking, and some passive smokers without recent smoke exposure might be missed.

Outcome and covariates

MAFLD

MAFLD was diagnosed as the presence of hepatic steatosis with at least one of the following[16,17]: 1) overweight or obesity (body mass index ≥25 kg/m^2); 2) diabetes mellitus; 3) at least two metabolic risk abnormalities (MRA). MRA consisted of 1) waist circumference ≥102 cm for men and ≥88 cm for women; 2) blood pressure ≥130/85 mmHg or specific drug treatment; 3) triglycerides ≥150 mg/dl; 4) HDL-cholesterol <40 mg/dl for men and <50 mg/dl for women; 5) prediabetes (fasting glucose 100-125 mg/dl or hemoglobin A1c 5.7%-6.4%; 6) homeostasis model assessment of insulin resistance (HOMA-IR) ≥2.5; 7) high-sensitive c-reactive protein (HS-CRP) >2 mg/L.

The original variables utilized for diagnosing MAFLD are presented in Table S6. It is crucial to handle missing data with care. For instance, when working with the seven variables in the MRA assessment, each variable receives a value of 1 if it meets the specified condition and 0 if it does not. MRA is deemed present if the sum of these values is greater than or equal to 2, indicating a metabolic disorder. However, MRA is usually misclassified as missing if any of the seven variables have a missing value.

In this context, two variables are essential to define MRA accurately: the number of missing values (A) and the sum of non-missing values (B). The following scenarios elucidate this: 1) B≥2 implies a diagnosis of MRA; 2) B=1, A=0 signifies a diagnosis of non-MRA; 3) B=1, A≥1 indicates a missing variable (NA); 4) B=0, A=1 or 0 results in a diagnosis of non-MRA; 5) B=0, A≥2 denotes a judgment of NA. Considering the number of missing variables is also pertinent when addressing issues related to obesity, type 2 diabetes, and metabolic disorders. The process procedure is the same as described above.

Table S6 Assessments of MAFLD characteristics

Original variable	Code	Assessment	Definition
Elastography exam status	LUAXSTAT	-	Complete —— valid<br>Partial —— invalid<br>Ineligible —— invalid<br>Not done —— invalid<br>Missing —— NA
Median CAP, decibels per meter (dB/m)	LUXCAPM	Median controlled attenuated parameter (CAP). This indicator is presented as a whole number, and the units for this measure are decibels per meter (dB/m).	100 to 400 — valid<br>Missing — NA
Median stiffness (E), kilopascals (kPa)	LUXSMED	Median liver stiffness (E). This indicator is presented with one digit to the right of the decimal ratio, and the units for this measure are kilopascals (kPa).	1.6 to 75 — valid<br>Missing — NA
*Body Mass Index (kg/m**2)*	BMXBMI	-	11.9 to 92.3 — valid<br>Missing — NA
Glycohemoglobin (%)	LBXGH	-	2.8 to 16.2 — valid<br>Missing — NA
Insulin (μU/mL)	LBXIN	-	0.71 to 512.5 — valid<br>Missing — NA
Waist Circumference (cm)	BMXWAIST	-	40 to 187.5 — valid<br>Missing — NA
Systolic blood pressure (mmHg)	BPXOSY1<br>BPXOSY2<br>BPXOSY3	-	52 to 225 — valid<br>Missing — NA
Diastolic blood pressure (mmHg)	BPXODI1<br>BPXODI2<br>BPXODI3	-	28 to 151 — valid<br>Missing — NA
Direct HDL-Cholesterol (mg/dL)	LBDHDD	-	5 to 189 — valid<br>Missing — NA
Triglyceride (mg/dL)	LBXTR	-	10 to 2684 — valid<br>Missing — NA
HS C-Reactive Protein (mg/L)	LBXHSCRP	-	0.011 — invalid (below lower detection limit)<br>0.011 to 246.86 — valid

Table S7 Assessments of covariates and other variables

Original variable	Code	Assessment	Definition
Interview/Examination status	RIDSTATR	Interview and examination status of the participant.	Interviewed only —— invalid<br>Both interviewed and MEC examined —— valid
Gender	RIAGENDR	-	Male —— valid<br>Female —— valid
Age in years at screening	RIDAGEYR	-	0 to 79 years —— valid<br>80 years of age and over —— 80
Race/Hispanic origin w/ NH Asian	RIDRETH3	Recode of reported race and Hispanic origin information, with Non-Hispanic Asian Category	Mexican American —— valid<br>Other Hispanic —— valid<br>Non-Hispanic White—— valid<br>Non-Hispanic Black —— valid<br>Non-Hispanic Asian —— valid<br>Other Race - Including Multi-Racial —— valid
Education level - Adults 20+	DMDEDUC2	What is the highest grade or level of school (you have/SP has) completed or the highest degree (you have/s/he has) received?	Less than 9th grade —— valid<br>9-11th grade—— valid<br>High school graduate —— valid<br>Some college or AA degree —— valid<br>College graduate or above —— valid<br>Refused —— NA<br>Don't Know —— NA<br>Missing —— NA
Marital status	DMDMARTZ	-	Married/Living with Partner —— valid<br>Widowed/Divorced/Separated —— valid<br>Never married —— valid<br>Refused —— NA<br>Don't Know —— NA<br>Missing —— NA
Family monthly poverty level category	INDFMMPC	-	Monthly PLI =1.30—— valid<br>1.30 < Monthly PLI = 1.85 —— valid<br>Monthly PLI >1.85 —— valid<br>Refused —— NA<br>Don't Know —— NA<br>Missing —— NA
Hepatitis B surface antigen	LBDHBG	-	Positive —— 1<br>Negative —— 0<br>Missing —— 0
Hepatitis C Antibody (confirmed)	LBDHCI	-	Positive —— 1<br>Negative —— 0<br>Negative Screening HCV Antibody —— 0<br>Positive HCV RNA —— 0<br>Missing —— 0

Mediators

Biological age

Biological age was proposed by Klemera and Doubal[18] _ENREF_16 based on eight biomarkers (C-reactive protein, serum creatinine, glycosylated hemoglobin, serum albumin, serum total cholesterol, serum urea nitrogen, serum alkaline phosphatase, and systolic blood pressure)[19]. R package ***bioage*** was used to calculate biological age in the analyses[20]. The values j and i represent the number of biomarkers and samples, respectively. The values k, q, and s are the regression slope, intercept, and root means squared error of a biomarker regressed on chronological age, respectively. The value r_j^2 represents the variance explained by regression of chronological age on biomarkers.

$$BA_E = \frac{\sum_{j=1}^{m}(x_j - q_j)\left(\frac{k_j}{s_j^2}\right)}{\sum_{j=1}^{m}\left(\frac{k_j}{s_j}\right)^2}$$

$$r_{char} = \frac{\sum_{j=1}^{m}\frac{r_j^2}{\sqrt{1-r_j^2}}}{\sum_{j=1}^{m}\frac{r_j}{\sqrt{1-r_j^2}}}$$

$$S_{BA}^2 = \frac{\sum_{j=1}^{n}\left((BA_{Ei} - CA_i) - \frac{\sum_{i=1}^{n}(BA_{Ei} - CA_i)}{n}\right)^2}{n} - \left(\frac{1 - r_{char}^2}{r_{char}^2}\right)\left(\frac{(CA_{max} - CA_{min})^2}{12m}\right)$$

$$Biological\ age = \frac{\sum_{j=1}^{m}(x_j - q_j)\left(\frac{k_j}{s_j^2}\right) + \frac{CA}{s_{BA}^2}}{\sum_{j=1}^{m}\left(\frac{k_j}{s_j}\right)^2 + \frac{1}{s_{BA}^2}}$$

Directed acyclic graph (DAG)

We select the covariates in the regression model using DAG[21]. We first included as many variables as possible in DAG[22]. After reviewing the extensive literature and validating the dataset of this study, we plotted the DAG by the DAGitty program (http://www.dagitty.net/dags.html). We finally only adjusted those covariates included in the minimal sufficient adjustment sets (MSAS)[23].

Statistical methods

E-value

The E-value is an alternative approach to sensitivity analyses for unmeasured confounding in observational studies that avoids making assumptions that, in turn, require subjective assignment of inputs for some formulas. Specifically, an E-value analysis asks the question: how strong would the unmeasured confounding have to be to negate the observed results? The E-value itself answers this question by quantifying the minimum strength of association on the risk ratio scale that an unmeasured confounder must have with both the treatment and outcome, while simultaneously considering the measured covariates, to negate the observed treatment–outcome association. If the strength of unmeasured confounding is weaker than indicated by the E-value, then the main study result could not be overturned to one of "no association" (i.e., moving the estimated risk ratio to 1.0) by the unmeasured confounder. E-values can therefore help assess the robustness of the main study result by considering whether unmeasured confounding of this magnitude is plausible. The E-value provides a measure related to the evidence for causality, hence the name "E-value"[24].

We used the online E-value calculator to calculate E-value (https://www.evalue-calculator.com/evalue/)[25,26]. The outcome type was set in OR (outcome prevalence >15%), and the estimate type was set in main effect (of 1 exposure). The point estimate of HEI-2015 is 0.827, thus, the E-value of HEI-2015 is 1.43.

Population attributable fraction (PAF)

Much statistical analysis seeks to identify associations between exposures and outcomes. The PAF is an epidemiologic measure widely used to assess the public health impact of exposures in populations. PAF is defined as the fraction of all cases of a particular disease or other adverse condition in a population that is attributable to a specific exposure; PAF equals (O − E)/O, where O and E refer to the observed number of cases and the expected number of cases under no exposure, respectively[27]. For a specific exposure, such as smoking, it is assumed that we created a cohort study exploring the association between smoke exposure and MAFLD, and the data are shown in the table.

**Smoke exposure**	**MAFLD**		**Population**	**Risk**
	**Yes**	**No**		
**Smoker**	500 (a)	9,500 (b)	10,000	0.05
**Non-smoker**	900 (c)	89,100 (d)	90,000	0.01
**Column totals**	1400 (a+c)	98,600 (b+d)	100,000	0.014

When the entire population is non-smokers, the proportion of MAFLD reduced is the PAF, which can be expressed by Equation 1, and the result is 28.6%. In addition, PAF can be calculated as Equation 2[28], where $\boldsymbol{RR}$ and $\boldsymbol{P_{pop}}$ can be calculated as Equations 3 and 4. It can be found that Equations 1 and 2 are entirely equivalent.

$$\boldsymbol{PAF = \frac{\left(a - (a+b)\frac{c}{c+d}\right)}{a+c}} \tag{1}$$

$$\boldsymbol{PAF = \frac{P_{pop}(RR-1)}{P_{pop}(RR-1)+1}} \tag{2}$$

$$\boldsymbol{RR = \frac{\frac{a}{(a+b)}}{\frac{c}{(c+d)}}} \tag{3}$$

$$\boldsymbol{P_{pop} = \frac{a+b}{a+b+c+d}} \tag{4}$$

PAFs can be calculated for continuous variables by dividing them into dichotomous variables based on the cut-off values. e.g., Sun et al. explored the association between $PM_{2.5}$ and esophageal cancer and found the PAF because of annual average $PM_{2.5}$ concentration ≥35 μg/m^3 was 23.3%[29]. The new method in the manuscript uses q% to group the population, with the q% of the population with a lower risk of disease as the control and the remaining (1-q)% of the population with a higher risk as the exposure to calculate the PAF[30]. Thus, the method yields PAFs corresponding to different q values.

We used ***PAF_calc_continuous*** function in ***graphPAF*** R package to calculate PAFs for continuous exposures[31]. The calculation method was set to D.

Ridge and Least Absolute Shrinkage and Selection Operator (LASSO) regression

Ridge regression and LASSO are two different types of penalty regression approaches[32]. These penalty regression approaches are constructed by adding penalty terms to the standard ordinary least squares (OLS) method. These methods are widely used in environmental epidemiology to decrease the influence of collinearity when environmental mixtures are used as exposures. There is a cost to everything, and obtaining a minor variance of coefficient estimates in the face of collinearity problems can also introduce a bias in the coefficient estimates (bias-variance tradeoff), i.e., coefficients will be shrunk towards zero.

Ridge regression is L2 regularization (penalizes sum of squared residuals) for the loss function of OLS, and the estimate of $\boldsymbol{\beta}$ is as follows, where $\boldsymbol{\lambda}$ is the penalty. As $\boldsymbol{\lambda}$ converges to 0, the $\boldsymbol{\beta}$ estimate converges to the OLS estimate; as $\boldsymbol{\lambda}$ converges to infinity, the $\boldsymbol{\beta}$ estimate converges infinitely to 0 but will not equal 0, i.e., ridge regression does not allow for variable selection. Unlike ridge regression, LASSO is L1 regularization (penalizes the sum of their absolute values) for the loss function of OLS. As $\boldsymbol{\lambda}$ increases, more and more $\boldsymbol{\beta}$ estimate equals 0. Thus, LASSO allows for variable selection.

$$\widehat{\boldsymbol{\beta}}_{ridge} = (\boldsymbol{X'X} + \boldsymbol{\lambda I})^{-1}(\boldsymbol{X'Y})$$

We used the R package ***glmnet*** to perform the ridge regression and LASSO analysis. We standardized the data and generated a range of $\boldsymbol{\lambda}$ from small to large in advance, assessing the accuracy of the models corresponding to different $\boldsymbol{\lambda}$ through a 10-fold cross-validation method. Finally, we chose the best cross-validated $\boldsymbol{\lambda}$ in ridge and LASSO regression analysis. The original ridge and LASSO regression results corresponded to one unit change of exposures. We demonstrated the coefficients corresponding to per IQR increase of continuous exposures to make the results more comparable.

Weighted Quantile Sum (WQS)

WQS is a statistical method developed for exposure to environmental mixtures[33]. The WQS obtains a score (weighted quantile sum for all exposure) by assigning weights to all exposures categorized into quartiles or more groups and then incorporates that score into the regression model, which in turn yields an effect estimation for mixture exposure.

The WQS regression model is as follows. $\boldsymbol{g}$ represents any monotonic, differentiable link function as in a generalized linear model. $\boldsymbol{\mu}$ represents the mean value. $\boldsymbol{\beta_0}$ is the intercept. $\boldsymbol{\omega_i}$ is the unknown weight for the ith component. $\boldsymbol{q_i}$ represents the quantile of component (e.g., for quartiles, $\boldsymbol{q_i}$ = 0, 1, 2, or 3 for values in the 1st, 2nd, 3rd, or 4th quartile, respectively). $\boldsymbol{C}$ is the number of exposures. $\sum_{i=1}^{C} \boldsymbol{\omega_i q_i}$ represents the weighted index for the set of c chemicals of interest, where $\sum_{i=1}^{C} \boldsymbol{\omega_i q_i} = 1$ and $\boldsymbol{0 \leq \omega_i \leq 1}$. $\boldsymbol{\beta_1}$ is the regression coefficient of the weighted quantile sum. $\boldsymbol{z}$ is a vector of covariates. $\boldsymbol{\varphi}$ is a vector of regression coefficients for the covariates.

$$\boldsymbol{g(\mu) = \beta_0 + \beta_1 \left(\sum_{i=1}^{C} \omega_i q_i \right) + z'\varphi}$$

We used the R package ***gWQS*** to conduct the WQS analysis. We classified 40% of the data as the training set and 60% as the validation set and performed bootstrap for 100 times. When dealing with three continuous exposures, q is set to 4, i.e., quantile.

When dealing with three categorical variable exposures, q is set to NULL, i.e., the original scale of the variable was used without quantile quantification, which gives the effect of changing the categorical variable by one unit on the original scale, i.e., the effect brought about by changing from unhealthy to healthy.

Quantile G-computation (QGC)

QGC is a new method for analyzing environmental mixtures which integrates G-computation based on WQS regression[34]. G-computation is a commonly used method in causal inference. Compared with WQS, QGC estimates the overall mixture effect with the same procedure but estimates the parameters of a marginal structural model rather than a standard regression in WQS. This way, under common assumptions in causal inference such as exchangeability, causal consistency, positivity, no interference, and correct model specification, this model will also improve the causal interpretation of the overall effect. In addition, the procedure also allegedly overcomes the assumption of uni-direction in WQS, which also means that QGC can estimate both positive and negative weights simultaneously.

We used the R package ***qgcomp*** to conduct the QGC analysis. The ***qgcomp.noboot*** function only needs to set the quartiles. When dealing with three continuous exposures, q is set to 4, i.e., quantile. When dealing with three categorical variable exposures, q is set to NULL, i.e., the original scale of the variable is used without quantile quantification, which gives the effect of changing the categorical variable by one unit on the original scale, i.e., the effect brought about by changing from unhealthy to healthy.

Subgroup analysis

We conducted subgroup analyses by stratifying the population into subpopulations. In each subpopulation, we computed the joint effect of the lifestyle exposome and subsequently compared these effects between the subgroups. Z-tests were employed to conduct statistical comparisons of the joint effects between subgroups. Z can be calculated by the following equation:

$$Z = \frac{|\beta - \beta_{ref}|}{\sqrt{(SE^2 + SE_{ref}^2)}}$$

We assessed the significance of the same lifestyle in different subgroups and different lifestyles in the same subgroups solely by comparing the magnitudes of the weights. No statistical tests were applied because these weights represented point estimates.

Because the OR is noncollapsible and WQS, unlike logistic regression, mandates the re-estimation of exposure weights within each subgroup before determining the joint effect, it is possible that the joint effect for the entire population might not be represented as a linear combination of the total effects observed in the subgroups. In other words, the population-wide effect may not fall within the range of the subgroup joint effects. For instance, in Table 3, the ORs for the entire population, individuals aged ≥60 years, and those aged <60 years are 0.772, 0.712, and 0.718, respectively.

Bayesian Kernel Machine Regression (BKMR)

BKMR is a new approach to studying mixtures introduced by Bobb and colleagues[35]. The health outcome is regressed on a flexible function of the mixture constituents specified using a kernel function. BKMR can be used to estimate mixture effects, identify pollutants responsible for observed mixture effects, and visualize exposure-response curves for single pollutants and interactions between pollutants. A general model for BKMR is

$$Y_i = h(z_{i1}, \ldots, z_{iM})^T + x_i^T\beta + \varepsilon_i$$

Where Y_i is the outcome, $(z_{i1}, \ldots, z_{iM})^T$ is a vector of M exposure variables, x_i^T is a vector of covariates, and $\varepsilon_i \sim N(0, \sigma^2)$. In the context of environmental mixtures $h(\cdot)$ typically characterizes a high-dimensional exposure-response function that may incorporate non-linearity and/or interaction among the mixture constituents. We used the R package ***bkmr*** and ***bkmrhat*** to conduct the BKMR analysis. The number of iterations was set to 100,000 in the main analysis and 1,0000 in sensitivity analyses.

In brief, compared with ridge regression and LASSO, WQS could not only estimate the weights (importance) of different exposures but also the effects of joint exposure. Compared with QGC, WQS is more widely used and based on fewer prerequisite statistical assumptions. BKMR can be used to check whether the exposure-response relationship is linear.

Casual mediation analysis

Assume that a given research sample with sample size N has one-dimensional exposure, mediator, and outcome variables. For each research subject $\boldsymbol{i} \in \mathbf{1}, \mathbf{2}, \dots, \boldsymbol{N}$ in the sample, let $\boldsymbol{A_i}$ be the exposure it receives. $\boldsymbol{M_i}$ is the mediator that is actually observed, and $\boldsymbol{C_i} = \boldsymbol{C_{1i}}, \boldsymbol{C_{2i}}, \boldsymbol{C_{3i}}, \dots, \boldsymbol{C_{pi}}$ is a series of covariates that have been collected. $\boldsymbol{Y_{i,a}}$ denotes the possible values of the outcome of the research subject i in the case of exposure $\boldsymbol{A} = \boldsymbol{a}$, and $\boldsymbol{M_{i,a^*}}$ denotes the possible values of subject i's mediator when exposure $\boldsymbol{A} = \boldsymbol{a}^*$, and it should be noted that $\boldsymbol{M_{i,a^*}}$ does not denote a certain fixed value, but rather denotes the mediator's potential value when $\boldsymbol{A} = \boldsymbol{a}^*$. $\boldsymbol{Y_{i,am}}$ denotes the possible values of subject i's outcome when exposure $\boldsymbol{A} = \boldsymbol{a}$ and the mediator $\boldsymbol{M} = \boldsymbol{m}$. $\boldsymbol{Y_{i,aM_{a*}}}$ denotes the potential value of the mediated outcome of subject i when exposure A = a and the mediator variable is at exposure $\boldsymbol{A} = \boldsymbol{a}^*$. $\boldsymbol{Y_{i,aM_{a*}}}$ is the so-called nested counterfactual outcome, which takes into account both the role of exposure (denoted by a), and through the mediated potential variable $\boldsymbol{M_{i,a^*}}$ the effect of exposure on it, which takes into account the exposure-induced change in the mediator and the subsequent effect on the outcome. When exposure is dichotomous, there will be four nested counterfactuals $\boldsymbol{Y_{i,1M_1}}, \boldsymbol{Y_{i,1M_0}}, \boldsymbol{Y_{i,0M_0}}, \boldsymbol{Y_{i,0M_1}}$ for each individual, and the target estimator will be defined by these nested counterfactual outcomes.

Target estimator

In mediation analysis, the most common target estimators include Controlled direct effect (CDE), Natural direct effect (NDE) and Natural indirect effect (NIE):

$$\boldsymbol{CDE} = \boldsymbol{E}[\boldsymbol{Y_{am}} - \boldsymbol{Y_{a^*m}}]$$

$$\boldsymbol{NDE} = \boldsymbol{E}\left[\boldsymbol{Y_{aM_a}} - \boldsymbol{Y_{a^*M_a}}\right]$$

$$NIE = E\left[Y_{aM_a} - Y_{aM_{a^*}}\right]$$

The CDE is the mean difference in the value of the outcome of exposure compared to no exposure if all subjects are controlled by some means to the same level m of the mediator variable. The NIE is the mean difference value of the outcome if the exposures are all at level a, but the mediator is at M_{a*} and M_a, respectively. Similarly, the NDE is the mean difference in the outcome if the exposures are at a and a^*, but the mediator is at M_a.

Statistical assumptions

Within this theoretical framework of counterfactuals, the identification of causal mediating effects is based on the following four main assumptions:

Hypothesis 1: There is no uncorrected confounder of the association between the exposure and outcome. Given the set of covariates C, the exposure assignment mechanism is independent of the potential outcome.

$$Y_{am} \perp A|\, C$$

Hypothesis 2: There is no unadjusted confounder of the association between exposure and mediator. Given the set of covariates C, the exposure allocation mechanism is independent of the potential mediator taking.

$$M_a \perp A|C$$

Hypothesis 3: There is no unadjusted confounder between the mediator and outcome. Given the set of covariates C and exposure $A = a$, the assignment of the mediator is independent of the potential outcome.

$$Y_{am} \perp M|\{A = a, C\}$$

Hypothesis 4: Besides the three non-confounding assumptions of hypotheses 1 to 3 above, identifying mediating effects requires fulfilling the so-called “Cross-worlds independence” assumption. This condition holds when no confounders are influenced by the exposure between the mediator and the outcome.

$$Y_{am} \perp M_{a^*}|C$$

After the above assumptions are met, causal mediation effects can be identified without making any assumptions about the model or parameter distributions, known as the mediation formula:

$$NDE = E\left[Y_{aM_{a^*}} - Y_{a^*M_{a^*}} \mid c\right] = \sum E[Y \mid a, m, c] - E[Y \mid a^*, m, c]P(m \mid a^*, c)$$

$$NIE = E[Y_{aM_a} - Y_{aM_{a^*}} \mid c] = \sum E[Y \mid a, m, c]P(m \mid a, c) - P(m \mid a^*, c)$$

Casual mediation analysis based on regression

Parametric regression-based causal mediation analysis is a generalisation of the traditional linear structure equation model mediation approach using the theoretical framework of causal mediation[36]. Its implementation steps are as follows:

Step 1: Fit a regression model for the mediator variable, choosing different model types depending on the type of mediator variable, e.g., fitting a general linear model if the mediator is a continuous variable or a logistic model if the mediator is a dichotomous variable.

$$E[M \mid A, C] = \beta_0 + \beta_1 A + \beta_2' C$$

Step 2: Choose the appropriate connection function to fit the outcome model according to the type of outcome variable, in which the product term of exposure and mediator can be added if there may be an interaction between exposure and mediator.

$$E[Y \mid A, M, C] = \theta_0 + \theta_1 A + \theta_2 M + \theta_3 AM + \theta_4' C$$

Step 3: Derive the analytical solution expression for the mediation effect based on the mediation formula, and then use the model parameters estimated in the first two steps to calculate the target estimate. The analytical solution expression is as follows when both the mediator and the outcome are continuous variables.

$$CDE = (\theta_1 + \theta_3 m)(a - a^*)$$

$$PNDE = \theta_1 + \theta_3(\beta_0 + \beta_1 a^* + \beta_2' c)(a - a^*)$$

$$TNDE = \theta_1 + \theta_3(\beta_0 + \beta_1 a + \beta_2' c)(a - a^*)$$

$$\boldsymbol{PNIE} = (\boldsymbol{\theta_2\beta_1} + \boldsymbol{\theta_3\beta_1 a^*})(\boldsymbol{a} - \boldsymbol{a^*})$$

$$\boldsymbol{TNIE} = (\boldsymbol{\theta_2\beta_1} + \boldsymbol{\theta_3\beta_1 a})(\boldsymbol{a} - \boldsymbol{a^*})$$

Step 4: Calculate the standard errors by the Delta method or Bootstrapping method to test the hypothesis of the analyses[37].

At a technical level, we introduced mediator variables to formulate an exposure-mediator-outcome model within Model 3 of the single-exposure analyses. Subsequently, we encompassed all variables within Model 3 to construct an exposure-mediator model, culminating in estimating the mediation effect. In this study, we used the ***mediate*** function from the ***mediation*** R package for causal mediation analysis[38]. ***robustSE*** was set to ***TRUE*** to obtain robust standard errors, and the number of simulations was set to 10,000. It is worth noting that the results need to be viewed cautiously as the present study was a cross-sectional study that could not guarantee sequencing between exposures, mediators, and outcome and had a limited sample size.

Supplementary figures

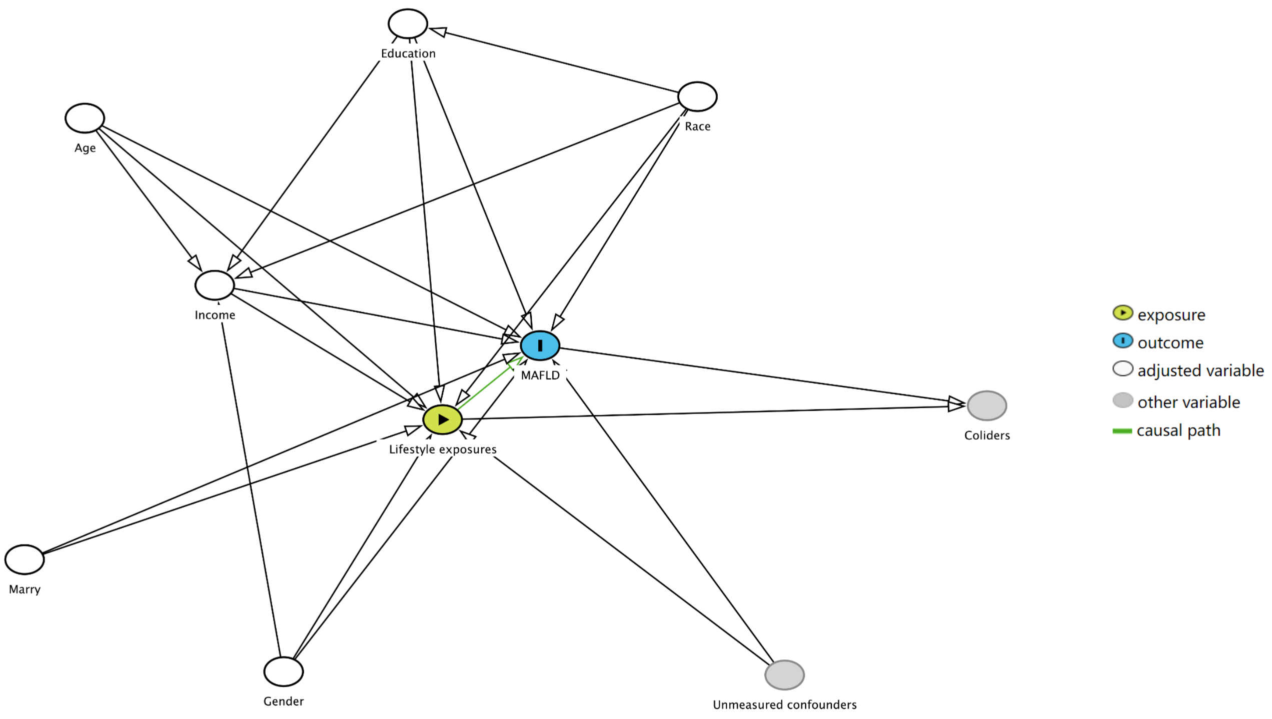

Figure S1 The directed acyclic graph (DAG) of this study

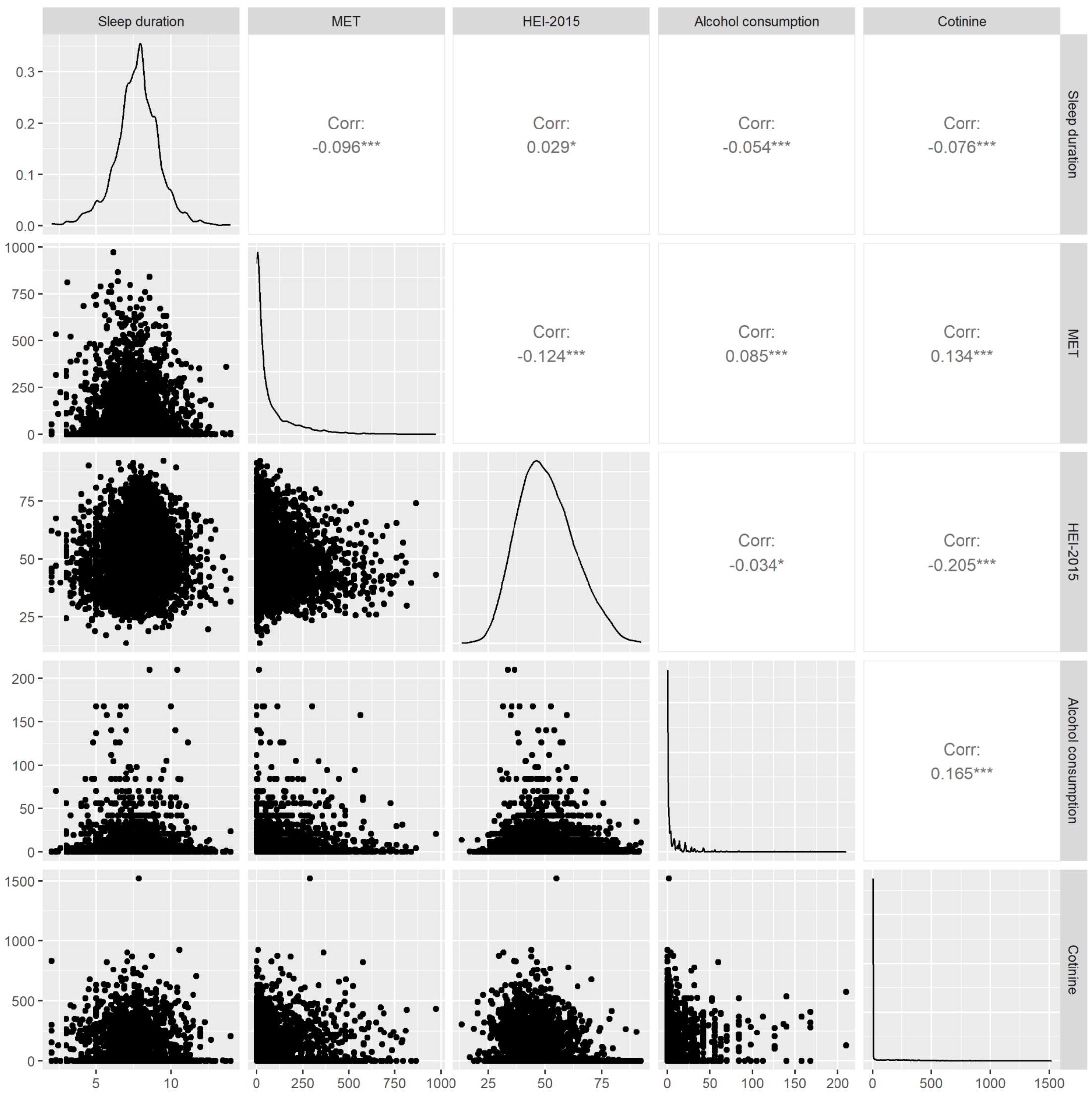

Figure S2 Distribution and correlation of five continuous exposures

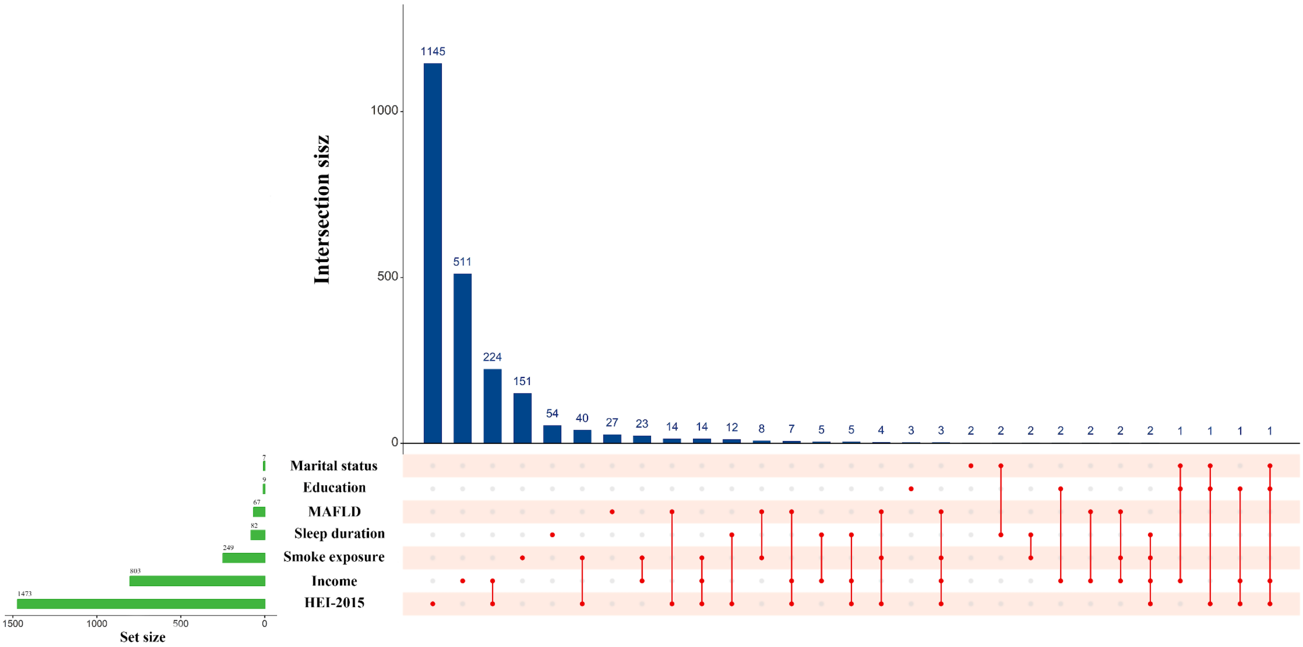

Figure S3 Distribution of missing data and its patterns

In the lower-left corner, we display the types of variables with missing data and the corresponding count of affected individuals. Meanwhile, in the lower-right corner, we present the various missing patterns, which indicate the presence of specific variables among the excluded individuals. Additionally, in the upper-right corner, we show the precise number of individuals conforming to each missing pattern.

Figure 1 illustrates that out of 7,270 participants, 67 were missing data for MAFLD, calculated as the sum of 27+14+8+7+4+3+2+2. Following the exclusion of MAFLD, individuals with missing exposure data must also be removed. These individuals exhibit a missing pattern that includes SLEEP, SMOKE, and HEI variables but excludes MAFLD. 1,682 participants were missing data for exposure, calculated as the sum of 1145+151+224+54+40+23+14+12+5+5+2+2+2+2+1+1+1+1. The remaining individuals to be excluded belong to the missing pattern that lacks variables SLEEP, SMOKE, HEI, and MAFLD. 519 participants were missing data for covariates, as determined by the sum of 511+3+2+2+1.

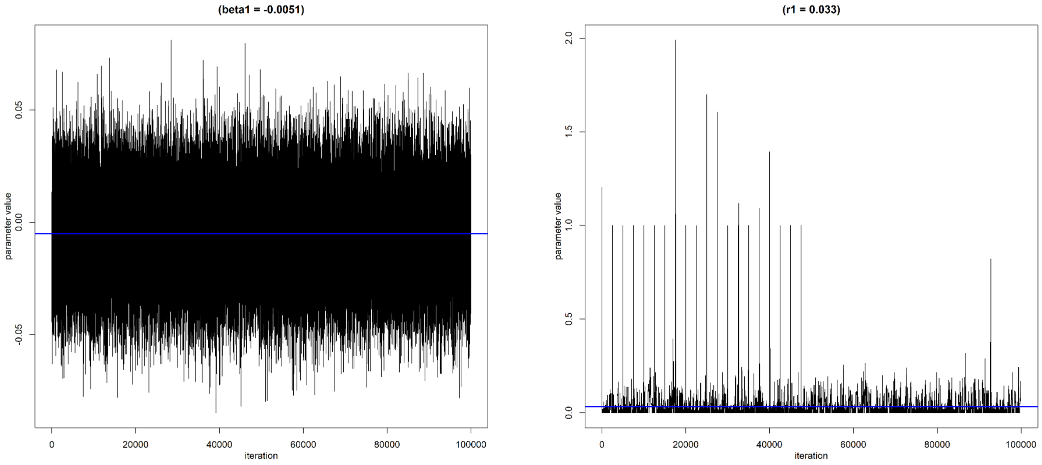

Figure S4 Traceplot of parameters in BKMR

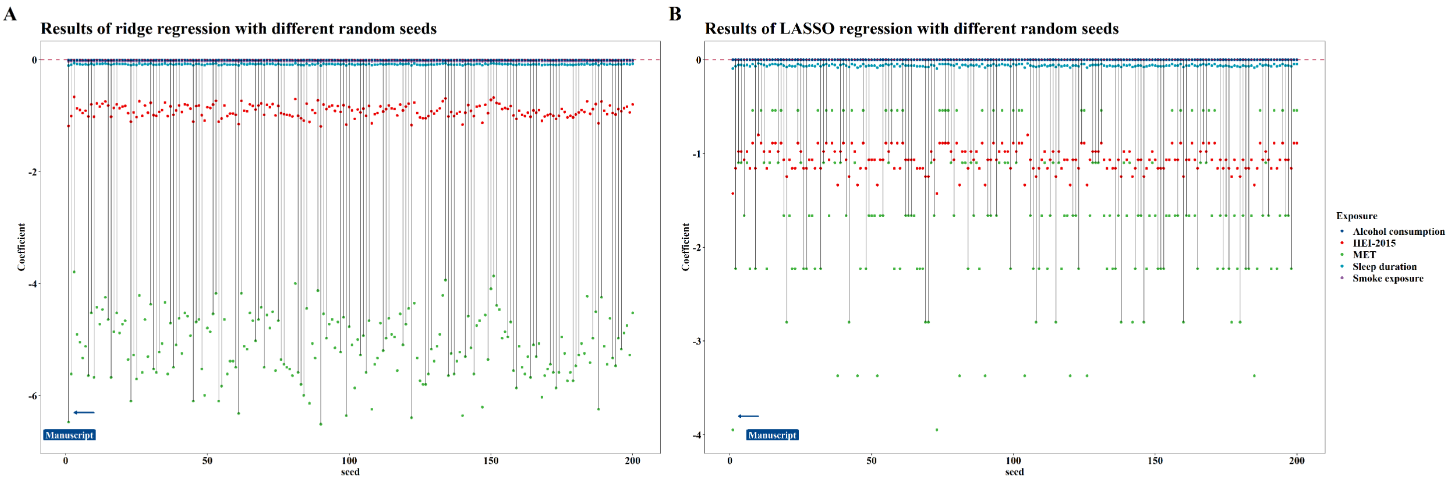

Figure S5 Sensitivity analysis of ridge and LASSO with different random seeds

The above models included age, gender, education, PLI, marital status, race, sleep duration, MET, alcohol consumption, smoke exposure, diet, and N=5,002. The coefficients shown in the figure are the exposure coefficients in the post-penalty model multiplied by the IQR of exposure.

In this figure, the horizontal coordinates represent different random seeds, the vertical coordinates represent the coefficients of each exposure variable when taking that seed, and the different colored points represent different exposure variables. It can be found that the relatively strong effects in Figure A are MET, HEI-2015, and Sleep duration, suggesting that these exposures are more important, and the relative relationship between exposures is not much related to the random seed, meaning that the results are relatively stable; in Figure B, the LASSO has selected the exposure variables, of which the coefficients of MET, HEI-2015, and Sleep duration are still non-0, suggesting that these exposures are more important, and the relative relationship between exposures is not much related to the random seeds, suggesting better stability of the results.

Abbreviations: LASSO: Least absolute shrinkage and selection operator; HEI: Healthy eating index; MET: Metabolic equivalent of task; OR: Odds ratio; CI: Confidence interval; PLI: Poverty level index.

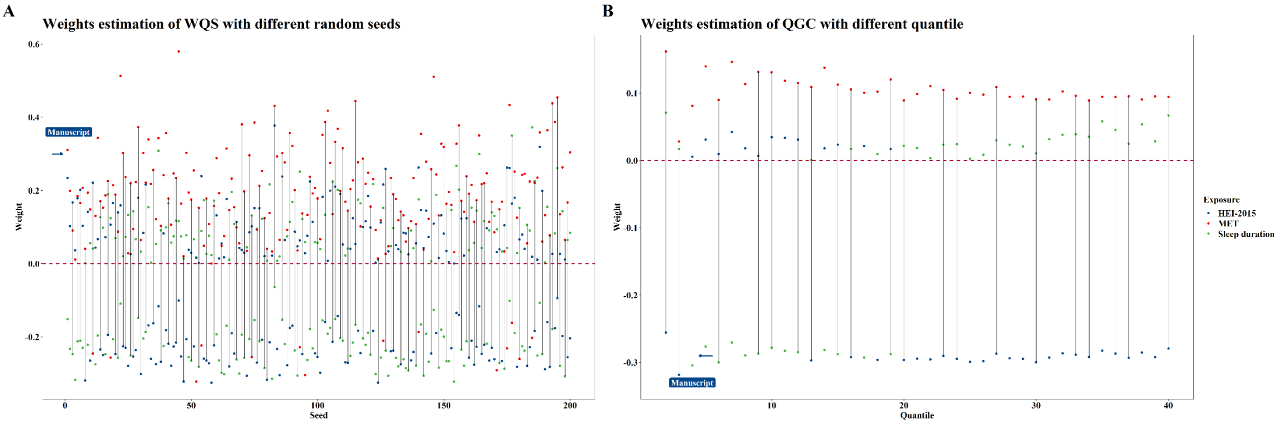

Figure S6 Sensitivity analysis of WQS (random seeds) and QGC (different quantile)

The above models included age, gender, education, PLI, marital status, race, sleep duration, MET, alcohol consumption, smoke exposure, diet, and N=5,002.

The horizontal coordinates in Figure A represent the different random seeds, the horizontal coordinates in Figure B represent the different quartiles taken, and the vertical coordinates represent all the weights. To easily distinguish the importance of different exposures, we have treated the weights as follows: the two larger weights are firstly subtracted from the smallest weight to save graphical space, preserving the relative relationship of the weights between these two exposures. Then, the smallest weight is shown as the reciprocal of its original value. Thus, in the above figure, the points below the horizontal coordinate represent the exposure corresponding to the smallest weight, and the points above the horizontal coordinate represent the exposure corresponding to the larger weight. The arrows point to the weighting results for the corresponding parameters in the manuscript. In both Figures A and B, the highest frequency of maximum weights occurs for MET, indicating the more robust results reported in the main text.

Abbreviations: HEI: Healthy eating index; MET: WQS: Weighted quantile sum; QGC: Quantile G-computation.

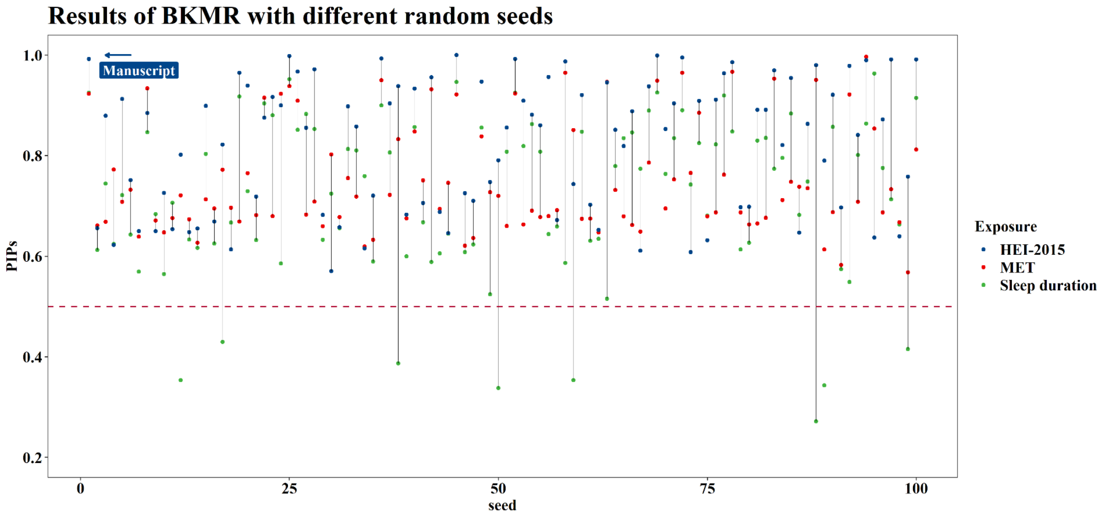

Figure S7 Sensitivity analysis of PIPs of BKMR with different random seeds

The above models included age, gender, education, PLI, marital status, race, sleep duration, MET, alcohol consumption, smoke exposure, diet, and N=5,002.

The horizontal coordinate represents the different random seeds, and the vertical coordinate represents all the PIPs results. The arrow points to the PIPs results for the corresponding parameters in the manuscript. The PIPs results of MET were all above 0.5 (a commonly used variable selection threshold[39]) and in the top two positions in 70% of the cases.

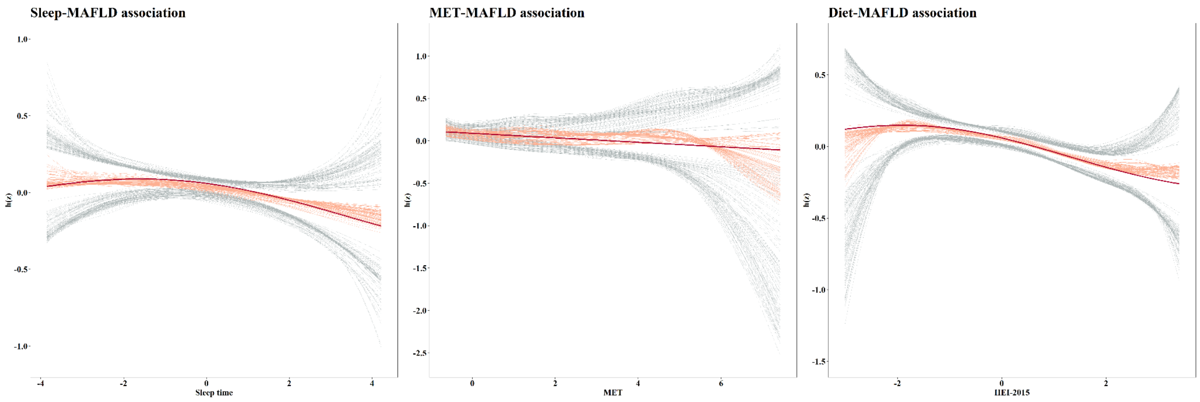

Figure S8 Singe exposure-MAFLD associations with different random seeds

Horizontal coordinates represent the centred continuous variables, and vertical coordinates represent the association of the variables with MAFLD. In each figure the brown solid line indicates the exposure response curve when different random seeds are taken, the red solid line indicates the exposure response curve in the manuscript, and the grey dashed line indicates the confidence interval when different random seeds are taken. It can be seen that the BKMR results are relatively robust.

Abbreviations: MAFLD: Metabolic- associated fatty liver disease; MET: Metabolic equivalent of task; HEI: Healthy eating index; OR: odds ratio; CI: confidence interval; PLI: Poverty level index.

Supplementary tables

Table S8 A comparison of the six statistical methods used in the manuscript

Method	Advantage	Limitation
Logistic regression	Widely accepted and easy to use	1. Cannot assess the overall mixture effect; 2. harder to handle complex interactions between exposures based on additivity assumptions; 3. affected by multicollinearity when multiple exposures are incorporated into the model
Ridge and LASSO regression	Reduce the effect of multicollinearity in effect estimations at the cost of a partial bias	1. Cannot assess the overall mixture effect; 2. more challenging to handle complex interactions between exposures based on additivity assumptions
WQS	1. The weights of the individual exposures were obtained and integrated into WQS, which in turn led to estimation of the effects of the mixtures; 2. has been adapted to four different cases: logistic, multinomial, Poisson and negative binomial regression; 3. fast operation speed	1. Makes an important assumption of uni-directionality (either positive or negative) of all exposures with respect to the outcome; 2. reduces statistical power caused by the need to split the dataset into training and validation sets
QGC	1. Estimates the effects of the mixtures and has a causal explanation; 2. overcomes the uni-directionality assumption in WQS and can estimate both positive and negative weights at the same time; 3. fast operation speed, and the code will yield an estimated running time	Requires fulfillment of causal inference assumptions
BKMR	1.Can be used to estimate mixture effects, identify pollutants which are responsible for observed mixture effects, visualize exposure-response curves for single pollutant and interactions between pollutants.2. The accompanying R package has a wealth of visualization capabilities	The program runs very slowly and is not recommended when the sample size is greater than 10,000

All these methods except BKMR require fulfillment of the linear assumption.

Abbreviations: LASSO: least absolute shrinkage and selection operator; WQS: weighted quantile sum; QGC: Quantile G-computation; BKMR: Bayesian Kernel Machine Regression.

Table S9 Statistical description of continuous exposure variables

Variable	Mean	SD	IQR	Max	P_{75}	Median	P_{25}	Min
Cotinine	54.8	128.0	3.3	1520.0	3.3	0.0	0.0	0.0
Sleep time	7.7	1.5	1.6	14.0	8.6	7.8	7.0	2.0
MET	76.3	120.6	93.3	972.0	96.0	25.3	2.7	0.0
Alcohol consumption	5.9	15.2	4.0	210.0	4.0	0.5	0.0	0.0
HEI-2015	50.5	12.3	17.0	92.3	58.6	49.4	41.5	13.5

Abbreviations: SD: standard deviation; IQR: inter quartile range; P_{75}: upper quartile; P_{25}: lower quartile; MET: Metabolic equivalent of task; HEI: Healthy eating index.

Table S10 Impact of missing data on population characteristics

Characteristic	Entire (N = 7,270)	Complete (N = 5,002)	*P*-value
**DEMOGRAPHIC CHARACTERISTICS**			
Age, years	50.48 (17.32)	50.43 (17.07)	0.890
Gender			0.158
female	3,689 (50.74%)	2,603 (52.04%)	
male	3,581 (49.26%)	2,399 (47.96%)	
Education			<0.001
Less than 9th grade	552 (7.60%)	263 (5.26%)	
9-11th grade	783 (10.78%)	507 (10.14%)	
High school graduate/GED or equivalent	1,739 (23.95%)	1,144 (22.87%)	
Some college or AA degree	2,357 (32.46%)	1,749 (34.97%)	
College graduate or above	1,830 (25.20%)	1,339 (26.77%)	
PLI			0.449
PLI=1.3	1,981 (30.63%)	1,485 (29.69%)	
1.3<PLI≤1.85	991 (15.32%)	756 (15.11%)	
PLI>1.85	3,495 (54.04%)	2,761 (55.20%)	
Marry status			0.462
Married/Living with Partner	4,239 (58.36%)	2,969 (59.36%)	
Never married	1,437 (19.79%)	949 (18.97%)	
Widowed/Divorced/Separated	1,587 (21.85%)	1,084 (21.67%)	
Race			<0.001
Mexican American	873 (12.01%)	563 (11.26%)	
Non-Hispanic Asian	878 (12.08%)	514 (10.28%)	
Non-Hispanic Black	1,923 (26.45%)	1,305 (26.09%)	
Non-Hispanic White	2,481 (34.13%)	1,890 (37.78%)	
Other Hispanic	757 (10.41%)	484 (9.68%)	
Other Race - Including Multi-Racial	358 (4.92%)	246 (4.92%)	
**SLEEP**			
Sleep duration, hours	7.74 (1.50)	7.72 (1.49)	0.878
Sleep duration level			0.456
Moderate	4,378 (60.91%)	3,080 (61.58%)	

Too long or short	2,810 (39.09%)	1,922 (38.42%)	
Sleep debt level			0.585
Low	5,369 (74.69%)	3,758 (75.13%)	
High	1,819 (25.31%)	1,244 (24.87%)	
Sleep difficulty			0.015
No	5,224 (71.91%)	3,495 (69.89%)	
Yes	2,041 (28.09%)	1,506 (30.11%)	
Daytime sleepiness			0.376
Low	5,438 (74.89%)	3,710 (74.19%)	
High	1,823 (25.11%)	1,291 (25.81%)	
Total sleep score			0.834
0	115 (1.60%)	81 (1.62%)	
1	631 (8.79%)	463 (9.26%)	
2	1,750 (24.38%)	1,244 (24.88%)	
3	2,563 (35.71%)	1,759 (35.18%)	
4	2,118 (29.51%)	1,453 (29.06%)	
Total sleep score level			0.591
Abnormal	5,059 (70.49%)	3,547 (70.94%)	
Normal	2,118 (29.51%)	1,453 (29.06%)	
**PHYSICAL ACTIVITY**			
MET, hour/week	76.42 (123.60)	76.34 (120.62)	0.108
MET level			0.092
Inactive	2,666 (36.67%)	1,760 (35.19%)	
Active	4,604 (63.33%)	3,242 (64.81%)	
**DIET**			
HEI-2015	50.58 (12.29)	50.47 (12.28)	0.66
DASH	26.70 (3.06)	26.70 (3.05)	0.962
MED	5.89 (0.94)	5.88 (0.94)	0.886
DII	1.63 (1.55)	1.63 (1.56)	0.916
HEI2015 level			0.611
Low-quality	2,899 (50.01%)	2,526 (50.50%)	
High-quality	2,898 (49.99%)	2,476 (49.50%)	
**ALCOHOL**			
Alcohol consumption, g/day	5.64 (14.80)	5.87 (15.17)	<0.001

Alcohol consumption level			0.403
Excessive	417 (5.74%)	305 (6.10%)	
Moderate	6,853 (94.26%)	4,697 (93.90%)	
**SMOKE**			
Smoking status			0.042
Never	4,281 (58.89%)	2,859 (57.16%)	
Former	1,692 (23.27%)	1,262 (25.23%)	
Current	1,297 (17.84%)	881 (17.61%)	
Secondhand smoke status			0.666
Yes	886 (12.19%)	618 (12.36%)	
No	6384 (87.81%)	4,384 (87.64%)	
Smoke exposure status			0.994
Yes	3,875 (53.30%)	2,761 (55.20%)	
No	3,395 (46.70%)	2241 (44.80%)	
**MAFLD ASSOCIATED VARIABLES**			
CAP, dB/m	264.48 (62.15)	265.50 (62.32)	0.401
LSM, kPa	5.80 (4.49)	5.87 (4.78)	0.634
BMI, kg/m^2	29.73 (7.17)	29.97 (7.24)	0.060
GLU, mg/dl	113.13 (37.53)	113.47 (38.01)	0.916
HBA_{1c}, %	5.84 (1.10)	5.85 (1.12)	0.822
Waist, cm	100.27 (16.63)	100.92 (16.75)	0.031
SBP, mmHg	124.61 (18.94)	124.28 (18.75)	0.346
DBP, mmHg	74.80 (11.44)	74.82 (11.24)	0.823
Triglyceride,	111.19 (98.76)	111.83 (106.22)	0.996
HDL	53.52 (15.88)	53.50 (15.83)	0.983
HOMA-IR	4.41 (9.32)	4.48 (9.50)	0.562
HS-CRP	3.99 (8.26)	3.92 (6.82)	0.244

Abbreviations: MAFLD: Metabolic- associated fatty liver disease; PLI: Poverty level index; MET: Metabolic equivalent of task; HEI: Healthy eating index; DASH: Dietary approaches to stop hypertension; MED: Mediterranean diet; DII: Mediterranean diet; CAP: Controlled attenuation parameter; LSM: Liver stiffness measure; GLU: Fasting glucose; HBA_{1c}: Hemoglobin A_{1c}; SBP: Systolic blood pressure; DBP: Diastolic blood pressure; HDL: High-density lipoprotein; HOMA-IR: Homeostasis model assessment of IR; HS-CRP: High- sensitive c- reactive protein; SD: standard deviation.

Table S11 Sensitivity analyses for sleep-MAFLD association

Model	Population	Exposure	Outcome	Covariate	OR	LCI	HCI	*P*
1	Manuscript [a]	Manuscript	Manuscript	Manuscript	0.883	0.826	0.944	<0.001
2	**Cirrhosis** [b]	Manuscript	Manuscript	Manuscript	0.892	0.836	0.952	0.001
3	**Imputation**	Manuscript	Manuscript	Manuscript	0.892	0.844	0.943	<0.001
4	Manuscript	**Dichotomous sleep duration**	Manuscript	Manuscript	0.958	0.845	1.084	0.494
5	Manuscript	**Dichotomous sleep debt**	Manuscript	Manuscript	0.700	0.579	0.846	<0.001
6	Manuscript	**Dichotomous sleep difficulty**	Manuscript	Manuscript	0.717	0.631	0.816	<0.001
7	Manuscript	**Dichotomous overly sleepy**	Manuscript	Manuscript	0.733	0.640	0.838	<0.001
8	Manuscript	**Dichotomous sleep scores(>=2)**	Manuscript	Manuscript	0.635	0.526	0.767	<0.001
9	Manuscript	**Dichotomous sleep scores(>=4)**	Manuscript	Manuscript	0.729	0.636	0.835	<0.001
10	Manuscript	Manuscript	**MAFLD (CAP>=248)**	Manuscript	0.857	0.804	0.913	<0.001
11	Manuscript	Manuscript	Manuscript	**Health status**	0.892	0.837	0.951	<0.001
12	Manuscript	Manuscript	Manuscript	**Insurance**	0.888	0.834	0.947	<0.001
13	Manuscript	Manuscript	Manuscript	**Hypertension**	0.895	0.838	0.956	0.001
14	Manuscript	Manuscript	Manuscript	**Diabetes**	0.900	0.824	0.982	0.018

[a]: manuscript: This part is the same as what is used in the manuscript.

The sample size in the manuscript is 5002. The exposure in the manuscript is sleep duration. The outcome in the manuscript is MAFLD (CAP >=285). Covariates used in the manuscript: age, gender, education, PLI, marry status, race, and other lifestyle variables.

[b]: Compared with the manuscript, the modified parts are bolded and underlined.

Table S12 Sensitivity analyses for PA-MAFLD association

Model	Population	Exposure	Outcome	Covariate	OR	LCI	HCI	*P*
1	Manuscript [a]	Manuscript	Manuscript	Manuscript	0.916	0.871	0.963	0.001
2	**Cirrhosis** [b]	Manuscript	Manuscript	Manuscript	0.921	0.876	0.969	0.002
3	**Imputation**	Manuscript	Manuscript	Manuscript	0.935	0.899	0.973	0.001
4	Manuscript	**Dichotomous MET (>=10)**	Manuscript	Manuscript	0.791	0.696	0.9	<0.001
5	Manuscript	**Dichotomous MET (>=25.3)**	Manuscript	Manuscript	0.769	0.679	0.87	<0.001
6	Manuscript	Manuscript	**MAFLD (CAP>=248)**	Manuscript	0.938	0.894	0.984	0.009
7	Manuscript	Manuscript	Manuscript	**Health status**	0.931	0.885	0.98	0.006
8	Manuscript	Manuscript	Manuscript	**Insurance**	0.917	0.872	0.965	0.001
9	Manuscript	Manuscript	Manuscript	**Hypertension**	0.915	0.869	0.963	0.001
10	Manuscript	Manuscript	Manuscript	**Diabetes**	0.904	0.842	0.971	0.005

[a]: manuscript: This part is the same as what is used in the manuscript.

The sample size in the manuscript is 5002. The exposure in the manuscript is MET. The outcome in the manuscript is MAFLD (CAP >=285). Covariates used in the manuscript: age, gender, education, PLI, marry status, race, and other lifestyle variables.

[b]: Compared with the manuscript, the modified parts are bolded and underlined.

Table S13 Sensitivity analyses for diet-MAFLD association

Model	Population	Exposure	Outcome	Covariate	OR	LCI	HCI	*P*
1	Manuscript [a]	Manuscript	Manuscript	Manuscript	0.827	0.756	0.904	<0.001
2	**Cirrhosis** [b]	Manuscript	Manuscript	Manuscript	0.831	0.758	0.911	<0.001
3	**Imputation**	Manuscript	Manuscript	Manuscript	0.818	0.742	0.901	0.001
4	Manuscript	**DASH**	Manuscript	Manuscript	0.781	0.716	0.852	<0.001
5	Manuscript	**MED**	Manuscript	Manuscript	0.829	0.747	0.919	<0.001
6	Manuscript	**DII**	Manuscript	Manuscript	1.163	1.061	1.275	0.001
7	Manuscript	**Dichotomous (q=0.5, 49.4)**	Manuscript	Manuscript	0.839	0.741	0.952	0.006
8	Manuscript	**Dichotomous (q=0.75, 58.6)**	Manuscript	Manuscript	0.789	0.707	0.880	<0.001
9	Manuscript	Manuscript	**MAFLD (CAP>=248)**	Manuscript	0.825	0.755	0.901	<0.001
10	Manuscript	Manuscript	Manuscript	**Health status**	0.855	0.78	0.936	0.001
11	Manuscript	Manuscript	Manuscript	**Insurance**	0.826	0.755	0.903	<0.001
12	Manuscript	Manuscript	Manuscript	**Hypertension**	0.810	0.738	0.889	<0.001
13	Manuscript	Manuscript	Manuscript	**Diabetes**	0.802	0.71	0.906	<0.001

[a]: manuscript: This part is the same as what is used in the manuscript.

The sample size in the manuscript is 5002. The exposure in the manuscript is HEI-2015. The outcome in the manuscript is MAFLD (CAP >=285). Covariates used in the manuscript: age, gender, education, PLI, marry status, race, and other lifestyle variables.

[b]: Compared with the manuscript, the modified parts are bolded and underlined.

Table S14 Sensitivity analyses for smoke-MAFLD association

Model	Population	Exposure	Outcome	Covariate	OR	LCI	HCI	*P*
1	Manuscript [a]	Manuscript	Manuscript	Manuscript	1.067	0.939	1.213	0.318
2	**Cirrhosis** [b]	Manuscript	Manuscript	Manuscript	1.104	0.969	1.258	0.137
3	**Imputation**	Manuscript	Manuscript	Manuscript	1.033	0.920	1.160	0.582
4	Manuscript	**Cotinine**	Manuscript	Manuscript	0.996	0.993	0.998	0.001
5	Manuscript	**Dichotomous smoke index**	Manuscript	Manuscript	1.020	0.894	1.169	0.737
6	Manuscript	**Dichotomous secondhand smoke index**	Manuscript	Manuscript	1.090	0.902	1.313	0.376
7	Manuscript	Manuscript	**MAFLD (CAP>=248)**	Manuscript	1.040	0.918	1.182	0.526
8	Manuscript	Manuscript	Manuscript	**Health status**	1.009	0.886	1.149	0.894
9	Manuscript	Manuscript	Manuscript	**Insurance**	1.070	0.941	1.126	0.301
10	Manuscript	Manuscript	Manuscript	**Hypertension**	1.042	0.914	1.189	0.536
11	Manuscript	Manuscript	Manuscript	**Diabetes**	1.110	0.931	1.324	0.244

[a]: manuscript: This part is the same as what is used in the manuscript.

The sample size in the manuscript is 5002. The exposure in the manuscript is dichotomous variable Incorporating active and passive smoking. The outcome in the manuscript is MAFLD (CAP >=285). Covariates used in the manuscript: age, gender, education, PLI, marry status, race, and other lifestyle variables.

[b]: Compared with the manuscript, the modified parts are bolded and underlined.

Table S15 Sensitivity analyses for alcohol-MAFLD association

Model	Population	Exposure	Outcome	Covariate	OR	LCI	HCI	*P*
1	Manuscript [a]	Manuscript	Manuscript	Manuscript	1.001	0.985	1.016	0.967
2	**Cirrhosis** [b]	Manuscript	Manuscript	Manuscript	1.003	0.986	1.019	0.753
3	**Imputation**	Manuscript	Manuscript	Manuscript	0.999	0.986	1.013	0.943
4	Manuscript	**Heavy alcohol consumption**	Manuscript	Manuscript	1.110	0.865	1.415	0.419
5	Manuscript	Manuscript	**MAFLD (CAP>=248)**	Manuscript	1.003	0.987	1.020	0.679
6	Manuscript	Manuscript	Manuscript	**Health status**	1.001	0.985	1.017	0.948
7	Manuscript	Manuscript	Manuscript	**Insurance**	1.001	0.985	1.017	0.892
8	Manuscript	Manuscript	Manuscript	**Hypertension**	0.997	0.981	1.013	0.683
9	Manuscript	Manuscript	Manuscript	**Diabetes**	1.001	0.978	1.025	0.917

[a]: manuscript: This part is the same as what is used in the manuscript.

The sample size in the manuscript is 5002. The exposure in the manuscript is average alcohol consumption per day. The outcome in the manuscript is MAFLD (CAP >=285). Covariates used in the manuscript: age, gender, education, PLI, marry status, race, and other lifestyle variables.

[b]: Compared with the manuscript, the modified parts are bolded and underlined.

Table S16 Sensitivity analyses for WQS using different parameters combinations

Model	q	Validation	B	Max	Sleep	MET	HEI	Beta	SE	OR 95 %CI	*P*
1	2	0	100	MET	0.317	0.416	0.267	-0.650	0.108	0.522 (0.423, 0.645)	<0.001
2	2	0.1	100	MET	0.297	0.470	0.233	-0.199	0.338	0.820 (0.422, 1.590)	0.556
3	2	0.2	100	MET	0.205	0.547	0.248	-0.353	0.226	0.702 (0.451, 1.094)	0.118
4	2	0.3	100	MET	0.242	0.536	0.222	-0.495	0.184	0.609 (0.425, 0.874)	0.007
5	2	0.4	100	MET	0.203	0.534	0.264	-0.610	0.159	0.544 (0.398, 0.742)	<0.001
6	2	0.5	100	MET	0.224	0.560	0.217	-0.494	0.138	0.610 (0.465, 0.800)	<0.001
7	2	0.6	100	MET	0.169	0.583	0.248	-0.459	0.124	0.632 (0.496, 0.805)	<0.001
8	2	0.7	100	MET	0.134	0.529	0.338	-0.483	0.117	0.617 (0.490, 0.776)	<0.001
9	2	0.8	100	MET	0.036	0.515	0.449	-0.450	0.103	0.637 (0.521, 0.780)	<0.001
10	2	0.9	100	MET	0.006	0.742	0.252	-0.346	0.085	0.707 (0.599, 0.835)	<0.001
11	3	0	100	MET	0.317	0.358	0.326	-0.422	0.068	0.656 (0.574, 0.750)	<0.001
12	3	0.1	100	MET	0.294	0.384	0.321	-0.225	0.221	0.799 (0.518, 1.231)	0.309
13	3	0.2	100	MET	0.224	0.440	0.336	-0.267	0.150	0.766 (0.571, 1.027)	0.075
14	3	0.3	100	MET	0.256	0.445	0.299	-0.395	0.123	0.674 (0.530, 0.857)	0.001
15	3	0.4	100	MET	0.242	0.475	0.283	-0.393	0.104	0.675 (0.551, 0.827)	<0.001
16	3	0.5	100	MET	0.238	0.520	0.243	-0.298	0.089	0.742 (0.624, 0.883)	0.001
17	3	0.6	100	MET	0.217	0.502	0.281	-0.282	0.082	0.754 (0.642, 0.886)	0.001
18	3	0.7	100	MET	0.156	0.549	0.295	-0.288	0.073	0.750 (0.650, 0.865)	<0.001
19	3	0.8	100	MET	0.073	0.651	0.276	-0.260	0.061	0.771 (0.684, 0.870)	<0.001
20	3	0.9	100	MET	0.018	0.867	0.114	-0.146	0.047	0.864 (0.788, 0.948)	0.002
21	4	0	100	MET	0.292	0.394	0.314	-0.341	0.049	0.711 (0.646, 0.782)	<0.001

Model	q	Validation	B	Max	Sleep	MET	HEI	Beta	SE	OR 95 %CI	*P*
22	4	0.1	100	MET	0.279	0.410	0.311	-0.179	0.155	0.836 (0.617, 1.133)	0.248
23	4	0.2	100	MET	0.223	0.455	0.323	-0.256	0.108	0.774 (0.627, 0.956)	0.017
24	4	0.3	100	MET	0.258	0.460	0.282	-0.324	0.087	0.723 (0.609, 0.858)	<0.001
25	4	0.4	100	MET	0.234	0.473	0.293	-0.330	0.074	0.719 (0.621, 0.832)	<0.001
26	4	0.5	100	MET	0.225	0.513	0.262	-0.257	0.064	0.773 (0.682, 0.876)	<0.001
27	4	0.6	100	MET	0.211	0.526	0.263	-0.259	0.058	0.772 (0.688, 0.865)	<0.001
28	4	0.7	100	MET	0.171	0.515	0.315	-0.282	0.054	0.755 (0.679, 0.839)	<0.001
29	4	0.8	100	MET	0.086	0.593	0.321	-0.264	0.047	0.768 (0.701, 0.842)	<0.001
30	4	0.9	100	MET	0.015	0.880	0.105	-0.141	0.034	0.869 (0.813, 0.928)	<0.001
31	5	0	100	MET	0.263	0.419	0.318	-0.272	0.043	0.762 (0.701, 0.828)	<0.001
32	5	0.1	100	MET	0.239	0.451	0.309	-0.097	0.140	0.908 (0.690, 1.195)	0.491
33	5	0.2	100	MET	0.186	0.483	0.331	-0.187	0.095	0.830 (0.689, 0.999)	0.048
34	5	0.3	100	MET	0.224	0.489	0.286	-0.261	0.078	0.771 (0.661, 0.898)	0.001
35	5	0.4	100	MET	0.194	0.528	0.278	-0.263	0.066	0.769 (0.676, 0.875)	<0.001
36	5	0.5	100	MET	0.174	0.580	0.246	-0.204	0.057	0.816 (0.730, 0.912)	<0.001
37	5	0.6	100	MET	0.156	0.586	0.258	-0.189	0.052	0.828 (0.748, 0.916)	<0.001
38	5	0.7	100	MET	0.107	0.560	0.333	-0.209	0.047	0.811 (0.740, 0.890)	<0.001
39	5	0.8	100	MET	0.046	0.584	0.370	-0.206	0.042	0.814 (0.750, 0.883)	<0.001
40	5	0.9	100	MET	0.009	0.840	0.151	-0.131	0.034	0.877 (0.821, 0.937)	<0.001
41	6	0	100	MET	0.283	0.398	0.320	-0.230	0.035	0.795 (0.743, 0.851)	<0.001
42	6	0.1	100	MET	0.262	0.422	0.317	-0.126	0.114	0.882 (0.706, 1.102)	0.270
43	6	0.2	100	MET	0.199	0.466	0.335	-0.149	0.077	0.862 (0.741, 1.002)	0.052
44	6	0.3	100	MET	0.236	0.469	0.296	-0.214	0.063	0.807 (0.713, 0.913)	0.001
45	6	0.4	100	MET	0.212	0.493	0.295	-0.220	0.054	0.802 (0.722, 0.891)	<0.001

**Model**	**q**	**Validation**	**B**	**Max**	**Sleep**	**MET**	**HEI**	**Beta**	**SE**	**OR 95 %CI**	***P***
46	6	0.5	100	MET	0.197	0.531	0.272	-0.173	0.047	0.841 (0.768, 0.921)	<0.001
47	6	0.6	100	MET	0.183	0.537	0.281	-0.159	0.042	0.853 (0.785, 0.927)	<0.001
48	6	0.7	100	MET	0.146	0.537	0.317	-0.175	0.039	0.840 (0.778, 0.906)	<0.001
49	6	0.8	100	MET	0.113	0.555	0.332	-0.189	0.035	0.827 (0.772, 0.887)	<0.001
50	6	0.9	100	MET	0.024	0.821	0.154	-0.107	0.027	0.898 (0.852, 0.947)	<0.001
51	7	0	100	MET	0.254	0.429	0.317	-0.193	0.029	0.825 (0.779, 0.873)	<0.001
52	7	0.1	100	MET	0.244	0.447	0.309	-0.089	0.094	0.915 (0.761, 1.101)	0.348
53	7	0.2	100	MET	0.192	0.490	0.318	-0.140	0.065	0.869 (0.766, 0.986)	0.030
54	7	0.3	100	MET	0.237	0.494	0.269	-0.185	0.053	0.831 (0.749, 0.921)	<0.001
55	7	0.4	100	MET	0.207	0.532	0.260	-0.187	0.044	0.829 (0.760, 0.904)	<0.001
56	7	0.5	100	MET	0.192	0.578	0.230	-0.142	0.038	0.868 (0.806, 0.935)	<0.001
57	7	0.6	100	MET	0.185	0.570	0.245	-0.139	0.035	0.870 (0.812, 0.931)	<0.001
58	7	0.7	100	MET	0.143	0.565	0.293	-0.153	0.032	0.858 (0.806, 0.913)	<0.001
59	7	0.8	100	MET	0.099	0.605	0.296	-0.157	0.029	0.855 (0.808, 0.904)	<0.001
60	7	0.9	100	MET	0.019	0.886	0.095	-0.085	0.021	0.919 (0.881, 0.958)	<0.001
61	8	0	100	MET	0.278	0.408	0.314	-0.172	0.025	0.842 (0.802, 0.884)	<0.001
62	8	0.1	100	MET	0.263	0.430	0.307	-0.075	0.080	0.928 (0.793, 1.085)	0.346
63	8	0.2	100	MET	0.206	0.469	0.326	-0.119	0.055	0.888 (0.797, 0.989)	0.031
64	8	0.3	100	MET	0.240	0.475	0.285	-0.157	0.045	0.855 (0.783, 0.933)	<0.001
65	8	0.4	100	MET	0.215	0.503	0.282	-0.164	0.038	0.849 (0.788, 0.914)	<0.001
66	8	0.5	100	MET	0.205	0.551	0.244	-0.125	0.033	0.883 (0.828, 0.941)	<0.001
67	8	0.6	100	MET	0.195	0.553	0.252	-0.121	0.030	0.886 (0.835, 0.939)	<0.001
68	8	0.7	100	MET	0.154	0.549	0.297	-0.135	0.028	0.874 (0.828, 0.922)	<0.001
69	8	0.8	100	MET	0.101	0.601	0.299	-0.137	0.025	0.872 (0.831, 0.915)	<0.001

Model	q	Validation	B	Max	Sleep	MET	HEI	Beta	SE	OR 95 %CI	*P*
70	8	0.9	100	MET	0.018	0.876	0.106	-0.073	0.018	0.929 (0.897, 0.963)	<0.001
71	9	0	100	MET	0.273	0.426	0.301	-0.153	0.023	0.858 (0.819, 0.898)	<0.001
72	9	0.1	100	MET	0.255	0.449	0.295	-0.050	0.077	0.951 (0.818, 1.105)	0.513
73	9	0.2	100	MET	0.199	0.489	0.312	-0.088	0.052	0.916 (0.827, 1.014)	0.090
74	9	0.3	100	MET	0.231	0.500	0.268	-0.137	0.043	0.872 (0.802, 0.948)	0.001
75	9	0.4	100	MET	0.200	0.532	0.268	-0.137	0.036	0.872 (0.813, 0.935)	<0.001
76	9	0.5	100	MET	0.187	0.570	0.243	-0.109	0.031	0.896 (0.844, 0.953)	<0.001
77	9	0.6	100	MET	0.178	0.572	0.249	-0.104	0.028	0.901 (0.852, 0.953)	<0.001
78	9	0.7	100	MET	0.134	0.568	0.298	-0.116	0.026	0.890 (0.846, 0.937)	<0.001
79	9	0.8	100	MET	0.088	0.609	0.303	-0.120	0.023	0.887 (0.847, 0.928)	<0.001
80	9	0.9	100	MET	0.018	0.858	0.124	-0.069	0.018	0.934 (0.901, 0.967)	<0.001
81	10	0	100	MET	0.265	0.413	0.322	-0.136	0.021	0.873 (0.838, 0.908)	<0.001
82	10	0.1	100	MET	0.244	0.439	0.317	-0.054	0.067	0.948 (0.831, 1.080)	0.421
83	10	0.2	100	MET	0.190	0.478	0.332	-0.094	0.045	0.910 (0.832, 0.995)	0.038
84	10	0.3	100	MET	0.229	0.485	0.286	-0.128	0.037	0.880 (0.818, 0.947)	0.001
85	10	0.4	100	MET	0.199	0.521	0.280	-0.128	0.032	0.880 (0.827, 0.936)	<0.001
86	10	0.5	100	MET	0.191	0.561	0.248	-0.099	0.027	0.906 (0.859, 0.956)	<0.001
87	10	0.6	100	MET	0.177	0.565	0.257	-0.094	0.025	0.910 (0.867, 0.956)	<0.001
88	10	0.7	100	MET	0.130	0.564	0.306	-0.103	0.023	0.902 (0.862, 0.943)	<0.001
89	10	0.8	100	MET	0.093	0.580	0.327	-0.112	0.021	0.894 (0.858, 0.931)	<0.001
90	10	0.9	100	MET	0.014	0.859	0.127	-0.061	0.016	0.940 (0.912, 0.970)	<0.001

Abbreviations: q: Quantile; B: Times of bootstrap; WQS: Weighted quantile sum; MET: Metabolic equivalent of task; HEI: health eating index; SE: standard error; OR: odds ratio; CI: confidence interval.

Table S17 Joint associations of sleep duration, diet, and physical activity exposure with MAFLD within different subgroups using QGC

Group	N [a]	Weight estimation			Joint association		*P* for subgroup comparisons
		Sleep	MET	HEI	OR 95% CI	*P*	
**Whole population** [b]	5,002	0.305	**0.385** [c]	0.310	0.710 (0.645, 0.782)	<0.001	—
**Gender** [d]							
Female	2,603	**0.419**	0.262	0.319	0.754 (0.644, 0.881)	<0.001	Ref
Male	2,399	0.122	**0.564**	0.314	0.720 (0.627, 0.825)	<0.001	0.664
**Age**							
≥60 years	1,765	0.221	**0.542**	0.237	0.701 (0.591, 0.832)	<0.001	Ref
<60 years	3,237	0.349	**0.463**	0.187	0.702 (0.619, 0.796)	<0.001	0.993
**PLI**							
PLI=1.3	1,485	**0.582**	0.345	0.074	0.773 (0.627, 0.954)	0.016	Ref
1.3<PLI≤1.85	756	0.079	**0.497**	0.424	0.712 (0.536, 0.947)	0.020	0.649
PLI>1.85	2,761	0.217	**0.455**	0.328	0.679 (0.596, 0.774)	<0.001	0.302
**Education**							
Less than 9th grade	263	0.133	0.320	**0.548**	0.793 (0.474, 1.325)	0.376	Ref
9-11th grade	507	0.236	**0.764**	-	0.862 (0.607, 1.224)	0.405	0.793
High school graduate/ GED or equivalent	1,144	**0.434**	0.429	0.137	0.640 (0.503, 0.815)	<0.001	0.459
Some college or AA degree	1,749	0.322	**0.408**	0.270	0.739 (0.628, 0.869)	<0.001	0.796
College graduate or above	1,339	0.231	**0.412**	0.357	0.586 (0.486, 0.708)	<0.001	0.279
**Race**							
Mexican American	563	**0.646**	0.341	0.012	0.727 (0.549, 0.962)	0.026	Ref
Non-Hispanic Asian	514	**0.552**	0.118	0.330	0.523 (0.382, 0.717)	<0.001	0.126
Non-Hispanic Black	1,305	0.294	**0.462**	0.244	0.773 (0.615, 0.973)	0.028	0.738
Non-Hispanic White	1,890	0.280	0.314	**0.406**	0.731 (0.626, 0.854)	<0.001	0.971
Other Hispanic	484	-	**0.689**	0.311	0.666 (0.460, 0.965)	0.032	0.714
Other Race - Including Multi-Racial	246	0.420	**0.580**	-	0.814 (0.507, 1.305)	0.391	0.687

[a]: N denotes the sample size in the model corresponding to each population.

[b]: Adjusted for age, gender, education, PLI, marry status, race, and all lifestyle variables.

[c]: The most important exposure in each model are bolded and underlined.

[d]: Adjusted all covariates in ***b*** except grouped variables, for example, gender here.

Abbreviations: MAFLD: Metabolic- associated fatty liver disease; MET: Metabolic equivalent of task; HEI: Healthy eating index; OR: odds ratio; CI: confidence interval; PLI: Poverty level index.

Table S18 Mediation effect of multiple mediators in lifestyle-MAFLD association

Exposure	Mediator	N [a]	ACME	*P*	ADE	*P*	TE	*P*	PM	*P*
Sleep duration	Bioage	4,830	-0.00108	0.154	-0.01601	0.000	-0.01709	0.000	0.06286	0.154
MET	Bioage	4,830	-0.00001	0.229	-0.00021	0.001	-0.00022	0.001	0.04224	0.229
HEI-2015	Bioage	4,830	-0.00012	0.199	-0.00271	0.000	-0.00282	0.000	0.04109	0.199
Sleep duration	Depression	4,884	-0.00045	0.051	-0.01532	0.001	-0.01577	0.001	0.02631	0.052
MET	Depression	4,884	0.00000	0.931	-0.00021	0.001	-0.00021	0.001	0.00047	0.931
HEI-2015 [b]	*Depression*	*4,884*	*-0.00008*	*0.042*	*-0.00241*	*0.000*	*-0.00249*	*0.000*	*0.03221*	*0.042*
Sleep duration	Health status	4,999	-0.00119	0.085	-0.01547	0.001	-0.01666	0.000	0.07080	0.086
MET	*Health status*	*4,999*	*-0.00004*	*0.000*	*-0.00016*	*0.005*	*-0.00021*	*0.001*	*0.20015*	*0.001*
HEI-2015	*Health status*	*4,999*	*-0.00046*	*0.000*	*-0.00199*	*0.001*	*-0.00245*	*0.000*	*0.18615*	*0.000*
Sleep duration	GLU	2,509	-0.00043	0.815	-0.00962	0.124	-0.01005	0.121	0.04853	0.770
MET	*GLU*	*2,509*	*-0.00006*	*0.008*	*-0.00021*	*0.012*	*-0.00026*	*0.001*	*0.21041*	*0.009*
HEI-2015	GLU	2,509	-0.00011	0.617	-0.00294	0.000	-0.00305	0.000	0.03641	0.617
Sleep duration	SBP	4,718	-0.00029	0.319	-0.01552	0.001	-0.01581	0.001	0.01715	0.320
MET	SBP	4,718	0.00000	0.249	-0.00021	0.000	-0.00022	0.000	0.01524	0.249
HEI-2015	*SBP*	4,718	*-0.00011*	*0.003*	*-0.00269*	*0.000*	*-0.00280*	*0.000*	*0.03853*	*0.003*
Sleep duration	*DBP*	4,718	*-0.00263*	*0.002*	*-0.01316*	*0.005*	*-0.01579*	*0.001*	*0.16601*	*0.003*
MET	*DBP*	4,718	*-0.00003*	*0.002*	*-0.00018*	*0.001*	*-0.00022*	*0.000*	*0.14910*	*0.002*
HEI-2015	*DBP*	4,718	*-0.00044*	*0.000*	*-0.00237*	*0.000*	*-0.00282*	*0.000*	*0.15583*	*0.000*
Sleep duration	BMI	4,977	-0.00837	0.000	-0.00756	0.063	-0.01593	0.001	0.52528	0.001
MET	BMI	4,977	-0.00011	0.000	-0.00010	0.055	-0.00021	0.000	0.53169	0.000
HEI-2015	*BMI*	*4,977*	*-0.00139*	*0.000*	*-0.00110*	*0.033*	*-0.00249*	*0.000*	*0.55775*	*0.000*
Sleep duration	Triglyceride	2,488	0.00077	0.588	-0.01128	0.079	-0.01050	0.112	-0.04551 [c]	0.664
MET	*Triglyceride*	*2,488*	*-0.00007*	*0.017*	*-0.00018*	*0.032*	*-0.00025*	*0.002*	*0.27942*	*0.019*
HEI-2015	Triglyceride	2,488	-0.00031	0.111	-0.00272	0.001	-0.00303	0.000	0.09495	0.111
Sleep duration	HDL	4,875	-0.00075	0.577	-0.01676	0.000	-0.01751	0.000	0.04265	0.577
MET	*HDL*	*4,875*	*-0.00006*	*0.000*	*-0.00015*	*0.009*	*-0.00021*	*0.000*	*0.30371*	*0.000*
HEI-2015	*HDL*	*4,875*	*-0.00090*	*0.000*	*-0.00172*	*0.002*	*-0.00262*	*0.000*	*0.34372*	*0.000*
Sleep duration	HBA_{1c}	*4,915*	*-0.00334*	*0.005*	*-0.01396*	*0.001*	*-0.01729*	*0.000*	*0.19240*	*0.005*
MET	HBA_{1c}	4,915	-0.00002	0.172	-0.00020	0.001	-0.00022	0.000	0.08992	0.172
HEI-2015	HBA_{1c}	4,915	-0.00020	0.207	-0.00238	0.000	-0.00258	0.000	0.07673	0.207
Sleep duration	CRP	4,840	0.00102	0.105	-0.01807	0.000	-0.01705	0.000	-0.05758	0.105
MET	*CRP*	*4,840*	*-0.00002*	*0.008*	*-0.00020*	*0.000*	*-0.00022*	*0.000*	*0.07726*	*0.009*
HEI-2015	*CRP*	*4,840*	*-0.00042*	*0.000*	*-0.00229*	*0.000*	*-0.00272*	*0.000*	*0.15358*	*0.000*
Sleep duration	ALT	4,846	0.00036	0.691	-0.01762	0.000	-0.01726	0.000	-0.02004	0.691
MET	ALT	4,846	-0.00002	0.084	-0.00020	0.001	-0.00022	0.001	0.10424	0.085

Exposure	Mediator	N	ACME	*P*	ADE	*P*	TE	*P*	PM	*P*
HEI-2015	ALT	4,846	-0.00007	0.536	-0.00269	0.000	-0.00276	0.000	0.02551	0.536
Sleep duration	AST	4,824	0.00068	0.023	-0.01828	0.000	-0.01760	0.000	-0.03669	0.023
MET	AST	4,824	0.00000	0.782	-0.00022	0.000	-0.00022	0.000	-0.00461	0.782
HEI-2015	AST	4,824	0.00004	0.268	-0.00274	0.000	-0.00270	0.000	-0.01455	0.268
Sleep duration	ALB	4,849	-0.00026	0.450	-0.01708	0.000	-0.01734	0.000	0.01418	0.451
MET	ALB	4,849	0.00000	0.460	-0.00022	0.001	-0.00022	0.000	0.01242	0.461
HEI-2015	*ALB*	*4,849*	*-0.00033*	*0.000*	*-0.00242*	*0.000*	*-0.00275*	*0.000*	*0.11974*	*0.000*
Sleep duration	ALP	4,846	0.00003	0.936	-0.01734	0.000	-0.01730	0.000	-0.00204	0.936
MET	ALP	4,846	0.00000	0.471	-0.00022	0.000	-0.00022	0.000	0.01590	0.471
HEI-2015	*ALP*	*4,846*	*-0.00027*	*0.000*	*-0.00249*	*0.000*	*-0.00275*	*0.000*	*0.09602*	*0.000*
Sleep duration	GGT	4,846	0.00021	0.728	-0.01750	0.000	-0.01729	0.000	-0.01078	0.728
MET	*GGT*	*4,846*	*-0.00002*	*0.004*	*-0.00021*	*0.001*	*-0.00022*	*0.000*	*0.06914*	*0.004*
HEI-2015	*GGT*	*4,846*	*-0.00020*	*0.001*	*-0.00256*	*0.000*	*-0.00276*	*0.000*	*0.07171*	*0.001*

[a]: The sample size used in the mediation analyses additionally excludes missing mediator compared to 5,002 in the manuscript. All the models were adjusted for age, gender, education, income, race, marital status, smoke exposure, alcohol consumption, HEI-2015, MET, and sleep duration.

[b]: When ACME, ADE, TE, and PM were all statistically significant, and ACME, ADE, and TE were protective, the row was marked in red italics as a meaningful mediating effect.

[c]: Since the total effect is inverse to the mediating effect, the PM here is negative and not meaningful.

Abbreviations: ACME: Average causal mediation effect; ADE: Average direct effect; TE: Total effect; PM: Proportion mediated; MET: Metabolic equivalent of task; HEI: Healthy eating index; GLU: Fasting glucose; SBP: Systolic blood pressure; DBP: Diastolic blood pressure BMI: Body mass index; HDL: High-density lipoprotein; HBA_{1C}: Hemoglobin A_{1c}; CRP: C-reactive protein; ALT: Alanine aminotransferase; AST: Aspartate aminotransferase; ALB: Albumin; ALP: Alkaline phosphatase; GGT: Gamma-glutamyl transferase.

Reference

1. Di H, Guo Y, Daghlas I, et al. Evaluation of Sleep Habits and Disturbances Among US Adults, 2017-2020. *JAMA network open* 2022; **5**(11): e2240788.
2. Chunnan L, Shaomei S, Wannian L. The association between sleep and depressive symptoms in US adults: data from the NHANES (2007-2014). *Epidemiol Psychiatr Sci* 2022; **31**: e63.
3. Shams-White MM, Pannucci TE, Lerman JL, et al. Healthy Eating Index-2020: Review and Update Process to Reflect the Dietary Guidelines for Americans,2020-2025. *J Acad Nutr Diet* 2023; **123**(9): 1280-8.
4. Reedy J, Lerman JL, Krebs-Smith SM, et al. Evaluation of the Healthy Eating Index-2015. *J Acad Nutr Diet* 2018; **118**(9): 1622-33.
5. Appel LJ, Moore TJ, Obarzanek E, et al. A clinical trial of the effects of dietary patterns on blood pressure. DASH Collaborative Research Group. *N Engl J Med* 1997; **336**(16): 1117-24.
6. Willett WC, Sacks F, Trichopoulou A, et al. Mediterranean diet pyramid: a cultural model for healthy eating. *The American journal of clinical nutrition* 1995; **61**(6 Suppl): 1402S-6S.
7. Shivappa N, Steck SE, Hurley TG, Hussey JR, Hébert JR. Designing and developing a literature-derived, population-based dietary inflammatory index. *Public Health Nutr* 2014; **17**(8): 1689-96.
8. Zhan JJ, Hodge RA, Dunlop AL, et al. Dietaryindex: A User-Friendly and Versatile R Package for Standardizing Dietary Pattern Analysis in Epidemiological and Clinical Studies. 2023: 2023.08.07.548466.
9. Zhan JJ. R package Dietaryindex. **https://github.com/jamesjiadazhan/dietaryindex** (accessed September 10 2023).
10. Institute NC. HEI Scoring Algorithm. **https://epi.grants.cancer.gov/hei/hei-scoring-method.html** (accessed September 10 2023).
11. Piercy KL, Troiano RP, Ballard RM, et al. The Physical Activity Guidelines for Americans. *Jama* 2018; **320**(19): 2020-8.
12. Ciardullo S, Muraca E, Cannistraci R, Perra S, Lattuada G, Perseghin G. Low 25 (OH) vitamin D levels are associated with increased prevalence of nonalcoholic fatty liver disease and significant liver fibrosis. *Diabetes Metab Res Rev* 2023; **39**(5): e3628.
13. Promotion NCfCDPaH. Excessive Alcohol Use. **https://www.cdc.gov/chronicdisease/resources/publications/factsheets/alcohol.htm** (accessed September 10 2023).
14. Shastri SS, Talluri R, Shete S. Disparities in Secondhand Smoke Exposure in the United States: National Health and Nutrition Examination Survey 2011-2018. *JAMA Intern Med* 2021; **181**(1): 134-7.
15. Verification SSoB. Biochemical verification of tobacco use and cessation. *Nicotine & Tobacco Research* 2002; **4**(2): 149-59.
16. Eslam M, Newsome PN, Sarin SK, et al. A new definition for metabolic dysfunction-associated fatty liver disease: An international expert consensus statement. *J Hepatol* 2020; **73**(1): 202-9.
17. Eslam M, Sanyal AJ, George J, et al. MAFLD: A Consensus-Driven Proposed Nomenclature

for Metabolic Associated Fatty Liver Disease. *Gastroenterology* 2020; **158**(7): 1999-2014.e1.

18. Klemera P, Doubal S. A new approach to the concept and computation of biological age. *Mech Ageing Dev* 2006; **127**(3): 240-8.
19. Chen L, Zhao Y, Liu F, et al. Biological aging mediates the associations between urinary metals and osteoarthritis among U.S. adults. *BMC Medicine* 2022; **20**(1): 207.
20. Parker DC, Bartlett BN, Cohen HJ, et al. Association of Blood Chemistry Quantifications of Biological Aging With Disability and Mortality in Older Adults. *The Journals of Gerontology: Series A* 2019; **75**(9): 1671-9.
21. Lipsky AM, Greenland S. Causal Directed Acyclic Graphs. *JAMA* 2022; **327**(11): 1083-4.
22. Tennant PWG, Murray EJ, Arnold KF, et al. Use of directed acyclic graphs (DAGs) to identify confounders in applied health research: review and recommendations. *Int J Epidemiol* 2021; **50**(2): 620-32.
23. Diemer EW, Hudson JI, Javaras KN. More (Adjustment) Is Not Always Better: How Directed Acyclic Graphs Can Help Researchers Decide Which Covariates to Include in Models for the Causal Relationship between an Exposure and an Outcome in Observational Research. *Psychotherapy and psychosomatics* 2021; **90**(5): 289-98.
24. Haneuse S, VanderWeele TJ, Arterburn D. Using the E-Value to Assess the Potential Effect of Unmeasured Confounding in Observational Studies. *Jama* 2019; **321**(6): 602-3.
25. Sensitivity Analysis in Observational Research: Introducing the E-Value. 2017; **167**(4): 268-74.
26. Mathur MB, Ding P, Riddell CA, VanderWeele TJ. Web Site and R Package for Computing E-values. 2018; **29**(5): e45-e7.
27. Mansournia MA, Altman DG. Population attributable fraction. 2018; **360**: k757.
28. Miettinen OS. Proportion of disease caused or prevented by a given exposure, trait or intervention. *American journal of epidemiology* 1974; **99**(5): 325-32.
29. Sun D, Liu C, Zhu Y, et al. Long-Term Exposure to Fine Particulate Matter and Incidence of Esophageal Cancer: A Prospective Study of 0.5 Million Chinese Adults. *Gastroenterology* 2023; **165**(1).
30. Ferguson J, Maturo F, Yusuf S, O'Donnell M. Population attributable fractions for continuously distributed exposures. 2020; **9**(1).
31. Ferguson J, O'Connell M. Estimating and displaying population attributable fractions using the R package graphPAF; 2023.
32. Friedman JH, Hastie T, Tibshirani R. Regularization Paths for Generalized Linear Models via Coordinate Descent. *Journal of Statistical Software* 2010; **33**(1): 1 - 22.
33. Carrico C, Gennings C, Wheeler DC, Factor-Litvak P. Characterization of Weighted Quantile Sum Regression for Highly Correlated Data in a Risk Analysis Setting. *J Agric Biol Environ Stat* 2015; **20**(1): 100-20.
34. Keil AP, Buckley JP, O'Brien KM, Ferguson KK, Zhao S, White AJ. A Quantile-Based g-Computation Approach to Addressing the Effects of Exposure Mixtures. *Environmental health perspectives* 2020; **128**(4): 47004.
35. Bobb JF, Valeri L, Claus Henn B, et al. Bayesian kernel machine regression for estimating the health effects of multi-pollutant mixtures. *Biostatistics* 2015; **16**(3): 493-508.
36. VanderWeele TJ, Vansteelandt S. Odds ratios for mediation analysis for a dichotomous outcome.

Am J Epidemiol 2010; **172 12**: 1339-48.

37. Valeri L, VanderWeele TJ. Mediation analysis allowing for exposure–mediator interactions and causal interpretation: Theoretical assumptions and implementation with SAS and SPSS macros. *Psychological methods* 2013; **18**(2): 137-50.
38. Sales AC. Review:mediation Package in R. 2017; **42**(1): 69-84.
39. Coker E, Chevrier J, Rauch S, et al. Association between prenatal exposure to multiple insecticides and child body weight and body composition in the VHEMBE South African birth cohort. *Environ Int* 2018; **113**: 122-32.
